# Dietary restriction protects from age-associated DNA methylation and induces epigenetic reprogramming of lipid metabolism

**DOI:** 10.1186/s13059-017-1187-1

**Published:** 2017-03-28

**Authors:** Oliver Hahn, Sebastian Grönke, Thomas M. Stubbs, Gabriella Ficz, Oliver Hendrich, Felix Krueger, Simon Andrews, Qifeng Zhang, Michael J. Wakelam, Andreas Beyer, Wolf Reik, Linda Partridge

**Affiliations:** 1 Max Planck Institute for Biology of Ageing, 50931 Cologne, Germany; 2 Cellular Networks and Systems Biology, CECAD, University of Cologne, Joseph-Stelzmann-Str. 26, Cologne, 50931 Germany; 3 Epigenetics Programme, The Babraham Institute, Cambridge, CB22 3AT UK; 4 Barts Cancer Institute, Queen Mary University of London, London, EC1M 6BQ UK; 5 Bioinformatics Group, The Babraham Institute, Cambridge, CB22 3AT UK; 6 Inositide Lab, The Babraham Institute, Cambridge, CB22 3AT UK; 7 Center for Molecular Medicine Cologne, University of Cologne, Cologne, 50931 Germany; 8 The Wellcome Trust Sanger Institute, Cambridge, CB10 1SA UK; 9 Department of Genetics, Evolution and Environment, Institute of Healthy Ageing, University College London, London, WC1E 6BT UK

## Abstract

**Background:**

Dietary restriction (DR), a reduction in food intake without malnutrition, increases most aspects of health during aging and extends lifespan in diverse species, including rodents. However, the mechanisms by which DR interacts with the aging process to improve health in old age are poorly understood. DNA methylation could play an important role in mediating the effects of DR because it is sensitive to the effects of nutrition and can affect gene expression memory over time.

**Results:**

Here, we profile genome-wide changes in DNA methylation, gene expression and lipidomics in response to DR and aging in female mouse liver. DR is generally strongly protective against age-related changes in DNA methylation. During aging with DR, DNA methylation becomes targeted to gene bodies and is associated with reduced gene expression, particularly of genes involved in lipid metabolism. The lipid profile of the livers of DR mice is correspondingly shifted towards lowered triglyceride content and shorter chain length of triglyceride-associated fatty acids, and these effects become more pronounced with age.

**Conclusions:**

Our results indicate that DR remodels genome-wide patterns of DNA methylation so that age-related changes are profoundly delayed, while changes at loci involved in lipid metabolism affect gene expression and the resulting lipid profile.

**Electronic supplementary material:**

The online version of this article (doi:10.1186/s13059-017-1187-1) contains supplementary material, which is available to authorized users.

## Background

Increasing frailty as result of declining organismal function characterizes aging, which is also the major risk factor for prevalent diseases of older people such as diabetes, cancer and neurodegenerative disorders [1, 2]. Environmental and genetic interventions can ameliorate the effects of aging, with nutrition, nutrient-sensing signaling networks and metabolism playing evolutionarily conserved roles [1, 3–5]. Dietary restriction (DR), in which food intake is reduced while avoiding malnutrition, extends lifespan in diverse model and non-model organisms [3, 6]. DR induces a remarkably broad-spectrum improvement in health and resistance to aging-related diseases in both rodents [3, 5] and rhesus monkeys [7]. In humans, too, short-term DR increases multiple markers of metabolic and cardiovascular health [8].

Experiments in laboratory model organisms have indicated that the somatotropic axis [9] and nutrient-sensing insulin/insulin-like growth factor/mTOR network mediate at least part of the increased health from DR [10–12]. Mediators such as altered proteostasis [13], mitochondrial activity [14] and various forms of stress resistance [15] may also be important. However, the precise mechanisms mediating the effects of DR remain elusive [1, 5, 6, 16] and are likely to be time-dependent [16, 17]. DR induces major and tissue-specific changes in gene expression [18–20], and these have implicated shifts in energy homeostasis, mitochondrial function and lipid metabolism [16, 17, 21–23] as important processes induced by DR to improve health during ageing. Lipid and fatty acid metabolism are emerging as particularly central candidates, as these were commonly identified in two large-scale meta-analyses of more than 30 transcriptome datasets of independent DR experiments and originating from various tissues [18, 19]. DR-induced activation of triglyceride synthesis and breakdown has also been observed in *Drosophila melanogaster*, and these processes have been shown experimentally to be essential for full lifespan extension [24].

Epigenetic modifications are important regulators of transcriptional networks, and are labile to both aging and dietary interventions. DNA methylation occurs primarily in the CpG dinucleotide and is a conserved and somatically heritable mark that is generally associated with transcriptional repression [25–27]. Age-associated changes of methylation include both hyper- and hypomethylation at specific genomic locations that differ between tissues [28, 29]. This age-related “epigenetic drift” has been described as decreasing methylation across the genome, accompanied by locally restricted hypermethylation, predominantly occurring within promoter regions and/or CpG islands (CGIs) [25, 29–33]. In contrast, other studies have reported global age-related hypermethylation in human skin and murine hematopoietic stem cells [34–36]. A recent analysis of high-throughput methylation data identified a set of age-sensitive DNA methylation sites in humans that together strongly predict chronological age (“epigenetic clock”) [37, 38], but that are also sensitive to ethnicity [39], biological age [38], and infectious and other diseases [40, 41]. Dietary interventions, including starvation and protein deprivation, can also alter patterns of DNA methylation, potentially in a long-lasting manner [42, 43], including transgenerationally [26, 44].

Dietary, genetic and pharmacological interventions that improve health during aging and extend lifespan induce long-lasting changes in gene expression that mediate their effects. Here we have asked if and how age-related DNA methylation, transcription and lipid composition dynamics of mouse liver are affected by DR. We have carried out systematic profiling of methylation by whole genome bisulfite sequencing (BS-seq), of the transcriptome by RNA sequencing (RNA-seq) and of lipid composition by lipidomics in aging mouse cohorts with ad libitum (AL) and DR feeding. Our work uncovers a general protective function of DR against age-related methylation changes, together with epigenetic reprogramming of lipid metabolism genes.

## Results

### Dietary restriction transcriptionally regulates enzymes involved in DNA methylation

**Fig. 1:**
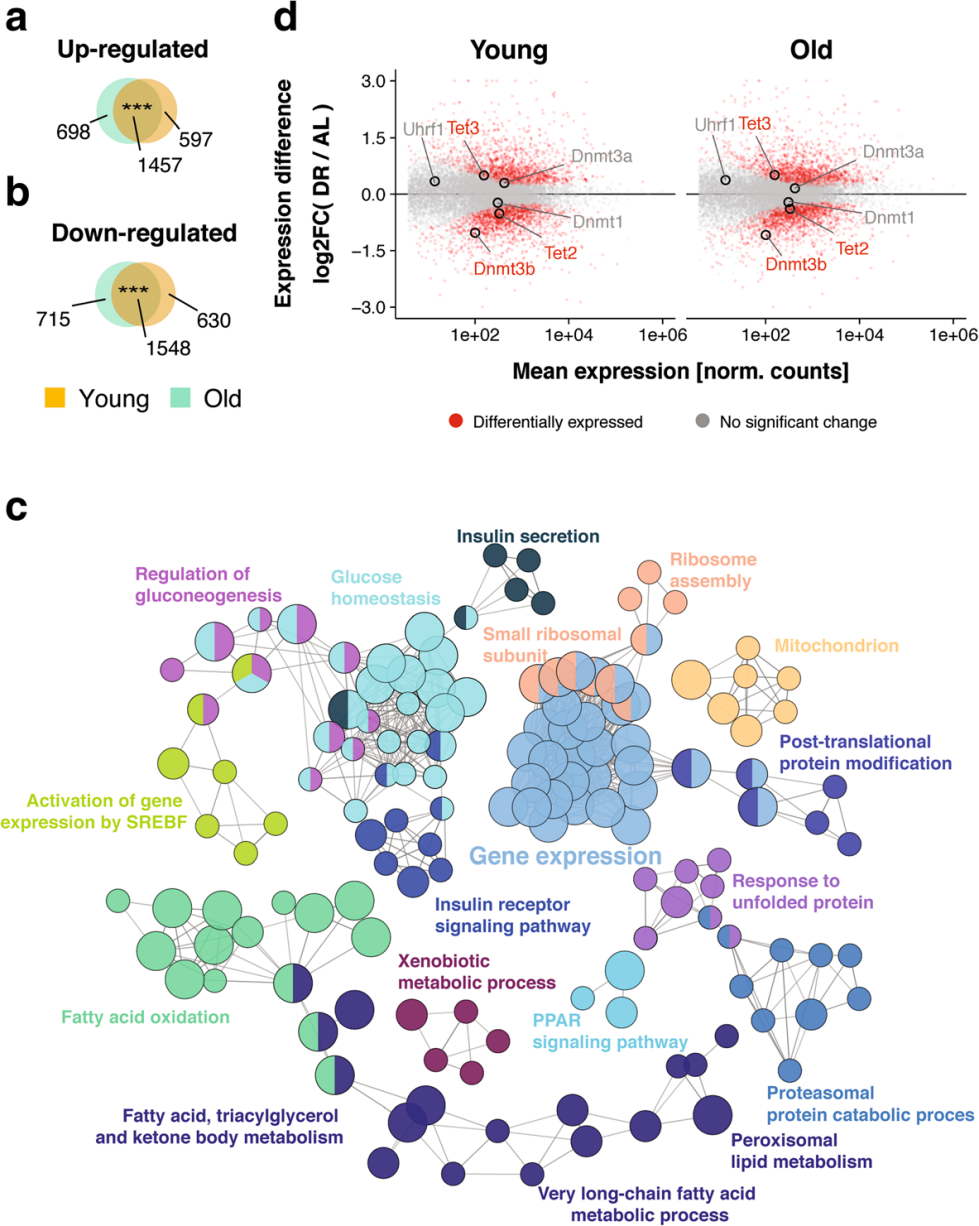
Transcriptome response to dietary restriction (*DR*) in the mouse liver. **a**, **b** Venn diagrams depicting the overlap of significantly upregulated (**a**) and downregulated (**b**) genes under DR relative to the ad libitum-fed (*AL*) control group at young and old age. *** *p* < 0.001, one-sided Fisher’s exact test. **c** Network representation of enriched gene ontology terms and pathways for genes differentially regulated under DR at both ages. Each node (*circles*) represents one term/pathway. Edges (*lines*) connect terms with similar gene sets (distance stands in inverse relationship with overlap); further functional clustering (*color*) was based on kappa statistics. Shown are representative terms per cluster. **d** MA-plots representing log_2_-transformed ratios of gene expression levels under DR and AL at both ages versus average expression on a logarithmic scale. Genes showing differential expression between the diets at both ages are marked in *red* (*n* = 3005). DNA de/methylation genes are highlighted

To investigate the effects of age and DR on gene expression and DNA methylation, we used females of the long-lived F1 hybrid mouse strain (C3B6F1), which responds to DR with a robust increase in lifespan [3]. To avoid effects on postnatal development, DR treatment was started in 12-week-old animals, restricting DR mice to 40% of the food intake of AL controls. The DR mice showed a 30% increase in median lifespan (Additional file 1: Figure S1A) with a reduced body weight compared to AL animals (Additional file 1: Figure S1B), consistent with previous observations [3, 5], thereby demonstrating the effectiveness of our DR regime. We collected liver samples from AL and DR animals at 5 months (young) and 26 months (old), and used RNA-seq to profile genome-wide transcriptional changes. We identified 4232 and 4418 differentially expressed genes (DEGs) between AL and DR in young and old animals, respectively, with 3005 DEGs in common (Fig. 1a, b), a highly significant overlap between young and old animals. DR-induced gene expression changes of selected candidate genes were validated by quantitative real-time polymerase chain reaction (qRT-PCR) on an independent set of mice (Additional file 1: Figure S2). Functional enrichment and clustering analysis for the overlapping DEGs highlighted fatty acid oxidation and lipid synthesis, insulin signaling and glucose homeostasis, protein catabolism, unfolded protein response, ribosome biogenesis, and xenobiotic metabolism (Fig. 1c; Additional file 2: Table S1), consistent with results of a meta-analysis of DR-induced expression changes in liver [18, 19]. The largest cluster contained genes involved in the regulation of gene expression, including *Dnmt3b*, which codes for one of the key enzymes involved in de novo DNA methylation (Fig. 1d) [45]. Additionally, *Tet2* and *Tet3*, which catalyze oxidation of 5-methylcytosine to 5-hydroxymethylcytosine and subsequent demethylation [45], were differentially regulated (Fig. 1d). DR may hence shape the DNA methylation landscape.

### DR protects from age-related changes in DNA methylation

We next asked whether DR affects the changes in DNA methylation that normally occur during aging. We profiled DNA methylation by genome-wide BS-seq of liver samples from young and old AL and DR mice. We focused our analysis on CpG methylation because levels of non-CpG methylation are extremely low in liver. The mammalian genome has local biases in CpG content [46, 47] and analyses of fixed-length DNA windows, or even predefined genomic regions, suffer from uneven data density, and therefore risk biasing detection of differences in methylation toward regions of high CpG density. We therefore made an unbiased comparison using a conservative quantification approach similar to [48] (summarized in Additional file 1: Figure S3A–D, for details see “Methods”), with a sliding, binning approach, with each bin covering 50 CpGs, and overlapping adjacent bins by 25 CpGs (Additional file 1: Figure S3A–C). This approach resulted in a set of 1,167,959 bins covering 29 million CpGs. For each bin we calculated a single methylation value averaged across CpGs ranging from 0% (no methylation) to 100%, (complete methylation). Genomic elements such as genes, promoters and CGIs were then mapped to bins. Median bin size was around 3500 bp (Additional file 1: Figure S3E), and we were able to detect well-established features such as low methylation in bins overlapping promoter CGIs compared to intragenic CGIs (Additional file 1: Figure S3F) [49, 50].

**Fig. 2:**
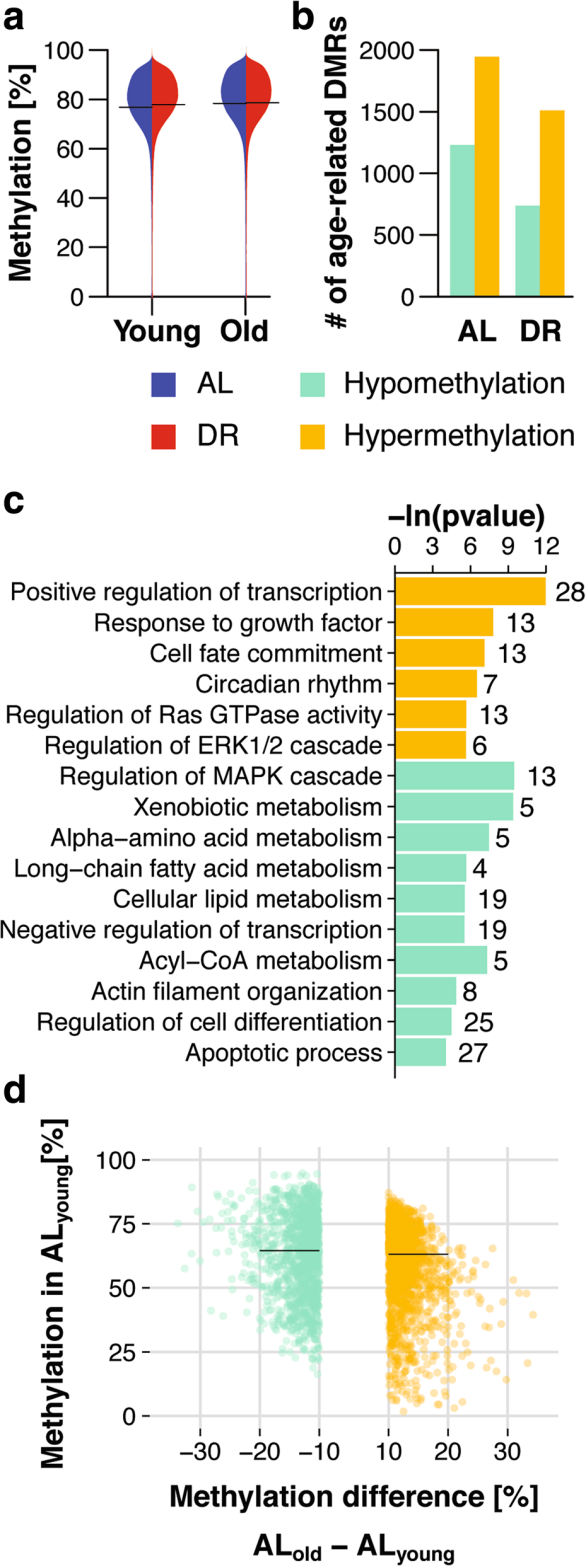
DNA methylation changes in the aging mouse liver. **a** Beanplot representation of global DNA methylation levels in the liver of young and old ad libitum-fed (*AL*) and dietary restriction (*DR*) mice (*n* = 1,167,959 bins). *Solid lines* represent group-wise means; density *curves* were scaled. **b** Number of significantly differentially methylated regions (*DMRs*) (*p* < 0.05, ±10% < DNA methylation difference) between young and old mice under AL and DR conditions. **c** Functional enrichment of age-related differentially hyper- and hypomethylated genes. **d** Density scatter plot of age-related DMRs indicating the magnitude of DNA methylation changes with respect to the methylation level in young AL mice. Lines indicate median of methylation levels for hypo- and hypermethylated DMRs

As expected, we found high levels of methylation genome-wide, with most bins having levels in the range of 70–95% (Fig. 2a). There were only subtle changes in global methylation with age or DR. In contrast to previously described age-related loss of DNA methylation [25], we observed a mild increase in global methylation with age (Fig. 2a), consistent with other recent genome-wide studies [34–36]. Although global changes were small, 3176 bins showed a significant methylation difference (adjusted *p* < 0.05, Chi-squared test; minimal required difference cutoff of 10%). Of these age-related differentially methylated regions (DMRs), 1945 gained and 1231 lost methylation (Fig. 2b), consistent with the small increase in global methylation with age. We next mapped age-related DMRs to gene bodies and identified 241 genes associated with hypomethylated (Additional file 3: Table S2) and 275 with hypermethylated DMRs (Additional file 4: Table S3). Among these, we identified several hypermethylated DMRs mapping to the body of the *Elovl2* gene. Hypermethylation of *Elovl2* is associated with aging in humans and mice [51–54]. Age-related hypermethylated genes showed functional enrichment for regulation of transcription, circadian rhythm, regulation of ERK and Ras signaling, and response to growth factor signaling (Fig. 2c; Additional file 5: Table S4). In contrast, age-related hypomethylated genes were involved in regulating the MAPK cascade, xenobiotic metabolism, and amino acid and lipid metabolism (Fig. 2c; Additional file 6: Table S5). Age-related changes in methylation were of moderate magnitude, in the range of 10–35% (Fig. 2d), consistent with previous studies [34–36].

**Fig. 3:**
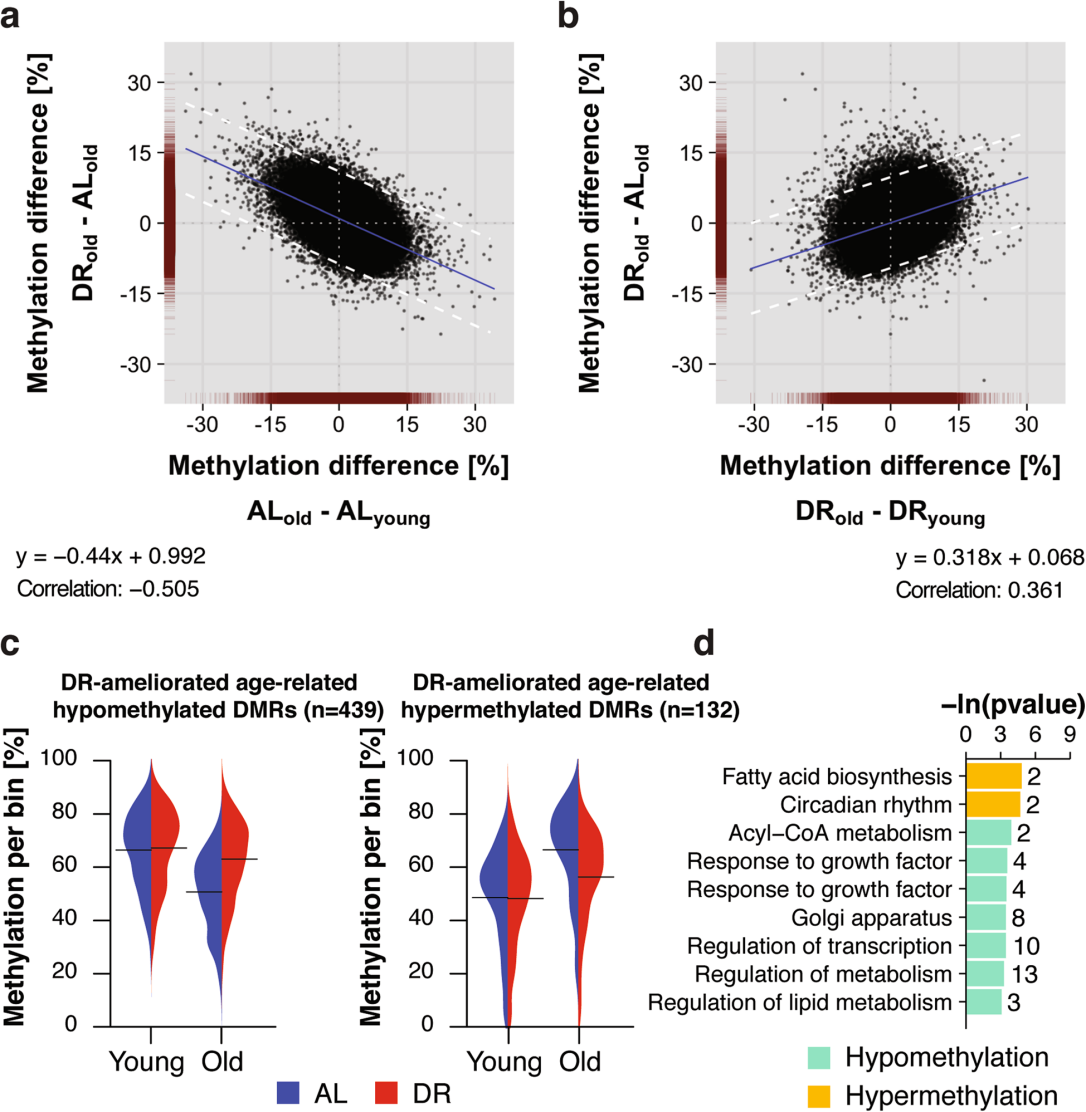
Dietary restriction (*DR*) attenuated age-related changes in DNA methylation. **a**, **b** Scatterplot comparison of bin-wise differences between diets at old age and age-related changes under ad libitum (*AL*) (**a**) and DR (**b**) conditions. Data density (*red*) is indicated on the axes. *Blue line* represents the linear regression line. *Dashed lines* mark the two standard deviations (2*σ) cutoff (±9.68% methylation difference). Formulas for linear regression and Pearson correlation coefficients are indicated. **c** Beanplot representation of methylation levels of 439 hypomethylated (*left panel*) and 132 hypermethylated (*right panel*) age-related differentially methylated regions (*DMRs*) ameliorated by DR in young and old mouse livers. *Solid lines* represent group-wise means; density curves are scaled. **d** Gene ontology and reactome enrichment of genes with age-related DMRs ameliorated by DR

In contrast to the 3176 age-related DMRs in AL animals, we identified only 2250 in DR animals, with 1512 hypermethylated and 738 hypomethylated with age (Fig. 2b). This lower number of age-related changes could indicate that DR retards age-related methylation changes. The null hypothesis is that the methylomes of DR and AL animals age at the same rate. We therefore took an unbiased approach, by comparing for each bin the difference in methylation between AL and DR at old age against the age-related change that occurred either under AL or DR (Fig. 3a, b). In striking contrast to the null model, there was a marked increase in scattering of bins during aging under DR conditions (Fig. 3b), caused by bins that showed age-related methylation differences in both conditions but with a greater magnitude in AL animals. The significantly decreased correlation and shallower slope of the relationship in the DR animals further confirmed this observation (Fig. 3a, b). To rule out any artifacts, we used permutation analysis of (1) treatment labels per bin, (2) across age within the same diet and (3) across diets within the same age (Additional file 1: Figure S4; for details see “Methods”), and the resulting distributions were clearly distinct from the data (Additional file 1: Figure S4). Thus, our analysis revealed global amelioration of age-related DMRs by DR. To characterize DMRs in which age-related changes were retarded by DR we used a statistical cutoff (Fig. 3a) to define 571 DMRs (Fig. 3b). Of these, 439 were hypomethylated and 132 hypermethylated with age. Beanplot representation of the data confirmed that the methylation of these DMRs changed with age in a diet-specific manner (Fig. 3c). DR-ameliorated age-related DMRs showed functional enrichment for genes involved in the regulation of transcription and in ketone, acetyl-CoA and long-chain fatty acid metabolism (Fig. 3d; Additional file 7: Table S6; Additional file 8: Table S7), with fewer genes affected than in the age-comparable dataset (Fig. 2c). DR thus retarded age-related changes in DNA methylation in specific functional categories of genes.

### Aging leads to demethylation of active regulatory regions and hypermethylation in repressed chromatin

We next asked whether age-related methylation changes correlated with chromatin state, as previously reported [55, 56]. We used publicly available histone modification chromatin immunoprecipitation sequencing (ChIP-seq) peaks from the ENCODE project, generated from the liver of 8-week-old male C57BL/6 mice [57]. Interestingly, age-related DMRs including the DR-ameliorated DMRs were strongly enriched for open chromatin histone marks, such as H3K9ac, H3K4me1 and H3K27ac [25, 27] (Additional file 1: Figure S5A), and this enrichment was more pronounced for age-related hypomethylated DMRs. Age-related hypomethylated DMRs were strongly enriched for distal and intragenic active enhancers and active promoters (Additional file 1: Figure S5B). Using the Cistrome platform [58, 59] we detected strong enrichment of age-related hypomethylated DMRs for a wide range of binding sites of DNA-binding proteins, most of which were transcription factors (Additional file 1: Figure S5C, D). Hence, age-related DMRs and particularly hypomethylated ones were strongly enriched in euchromatic regions of the genome, suggesting a role of chromatin accessibility in the methylation dynamics.

Bivalent chromatin domains are characterized by both activating H3K4me3 and repressive H3K27me3 modifications, and have been associated with age-related hypermethylation in humans and mice [33, 60]. We used indexed chromatin immunoprecipitation (co-ChIP) data (from 8-week-old female C57BL/6 J mice) that allow the detection of co-occurrences of H3K4me3 and H3K27me3 on the same nucleosome [61] to identify bivalent chromatin domains. We found a significant enrichment of age-related hypermethylated DMRs for bivalent CGIs (Additional file 1: Figure S5B), with 77 DMRs mapping onto 64 bivalent CGIs (Additional file 9: Table S8), exemplified by the Nol3 promoter CGI (Additional file 1: Figure S6A). Bivalent CGIs were also enriched among DR-ameliorated age-related DMRs, with 14 DMRs mapping onto seven bivalent CGIs (Additional file 1: Figure S5B). Age-related hypermethylated DMRs were also enriched for repressors, defined by H3K27me3 modifications (see “Methods” for details; Additional file 1: Figure S5B). Notably, DR-ameliorated age-related DMRs were different in that they were not enriched for repressive promoter chromatin (Additional file 1: Figure S5B). Future studies profiling chromatin marks in DR will uncover whether this indicates chromatin alterations brought about by DR or altered coupling between DNA methylation and histone marks.

Transposable elements have been implicated in age-related loss of genome integrity [43, 62]. However, transposons were under-represented among hyper- and hypomethylated DMRs (Additional file 1: Figure S7A, B). Thus, our data do not support a major role of DNA methylation in age-related loss of genome integrity mediated by transposable elements.

### Age-related differential methylation is not globally associated with changes in gene expression

DNA methylation of promoters and gene bodies can be associated with transcription although the links are often context-dependent [25, 49, 56, 63, 64]. A clear association between changes in DNA methylation and gene expression during aging has not been reliably established [25, 36, 65], with a possible exception of distal enhancer regions in mouse beta cells [66]. Because age-related DMRs showed a highly significant enrichment over genes (Additional file 1: Figure S8A), we asked whether they were associated with changes in gene expression in mouse liver. We selected genes with at least two age-related DMRs mapping to the gene body, including the transcription start site, and plotted age-related methylation differences and respective expression changes (Additional file 1: Figure S8B, C). We did not detect any significant association under either AL (Additional file 1: Figure S8B) or DR conditions (Additional file 1: Figure S8C). Similar results were obtained when the analysis was limited to genes that showed significant gene expression changes with age (red data points in Additional file 1: Figure S8B, C) and to DMRs that significantly changed with age under DR conditions (Additional file 1: Figure S8D, E). Hence, there is no global link between age-related methylation and transcription dynamics. However, in the next section we identify specific groups of genes in which such links were modified by age and diet.

### DR induces gene body methylation and associated transcriptional changes during aging

**Fig. 4:**
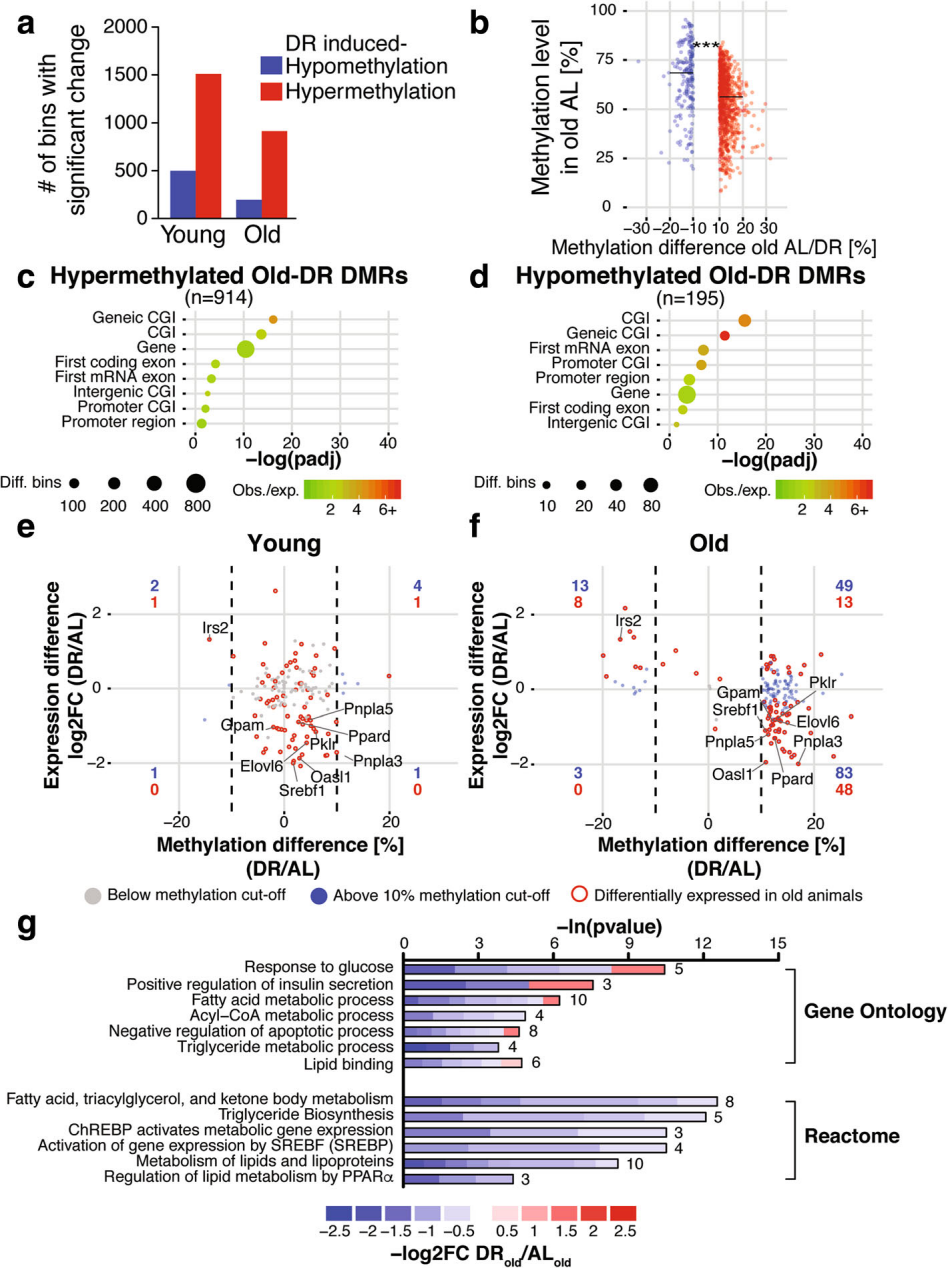
DNA methylation changes in the mouse liver in response to dietary restriction (*DR*). **a** Number of significantly (*p* < 0.05) differentially methylated regions (*DMRs*) (DNA methylation difference > ±10%) between ad libitum-fed (*AL*) and DR at young and old age. **b** Density scatter plot indicating the magnitude of DNA methylation changes between diets with respect to the methylation level of old AL animals. *Lines* indicate median of methylation levels for hypo- and hypermethylated DMRs. Average DNA methylation levels were significantly different between hypo- and hypermethylated Old-DR DMRs. *** *p* < 0.001, Wilcoxon-rank-sum test. **c**, **d** Enrichment analysis of hyper- (**c**) and hypomethylated (**d**) Old-DR DMRs over genomic elements. **e**, **f** Scatterplot of expression differences versus methylation differences of Old-DR DMRs in young (**e**) and old (**f**) animals. *Dashed lines* indicate DNA methylation cutoff of > ±10%. There was no significant correlation between DNA methylation and gene expression in young animals (Fisher’s test *p* = 1). In contrast, DNA methylation and gene expression were significantly negatively correlated in old animals (Fisher’s test *p* <0.001, Pearson correlation −0.387 for all genes; *p* <0.001, Pearson correlation −0.556 for differentially expressed genes). Number of differentially methylated genes in each quadrant is indicated in *blue* and *red*, for all genes and differentially expressed genes, respectively. **g** Gene ontology and reactome enrichment of genes with a negative correlation of gene expression and methylation. Lengths of bars represent negative log-transformed, adjusted *p* values using Fisher’s exact enrichment test. Cells indicate log_2_-fold changes (*log2FC*) between AL and DR per gene. *CGI* CpG island

DR can induce diet-specific gene expression programs independent of age effects [17, 19], and we therefore examined how DR affected DNA methylation at young and old age. We identified 2008 and 1109 diet-induced DMRs in young and old animals, respectively (Fig. 4a). In young animals, 1509 were hypermethylated and 499 hypomethylated, while in old animals 914 were hypermethylated and 195 hypomethylated. DR therefore predominantly caused hypermethylation at both ages (Fig. 4a). Diet-induced methylation changes were in the range of 10–30% (Fig. 4b), similar to the magnitude of age-related methylation changes (compare Fig. 2d).

We identified significantly more dietary DMRs at young age (2008) than at old age (1109) (Fig. 4a). There was only a small subset of DMRs that were differentially methylated in both young and old animals (highlighted red in Additional file 1: Figure S9A), indicating that long-term DR treatment induces methylation changes that are different from the apparently transient changes seen in young DR animals.

In young animals there was weak enrichment of DR DMRs over genic elements, including genes (Additional file 1: Figure S9B, C), and there was no significant correlation between differential methylation and transcription (Additional file 1: Figure S9D, E). Notably, however, in old animals there was a strong enrichment of DR DMRs in the gene body (Fig. 4c, d). Genes with DR-induced methylation differences in the gene body were enriched for fatty acid, triglyceride and ketone body metabolism-related gene ontology (GO) terms (Additional file 1: Figure S10A; Additional file 10: Table S9; Additional file 11: Table S10; Additional file 12: Table S11; Additional file 13: Table S12). Intriguingly, in old animals DR also resulted in a significant negative correlation between DNA methylation and gene repression on a larger scale (Fig. 4f). Hence, 56 genes showed an inverse relationship between gene expression and DNA methylation (Fig. 4f; Additional file 11: Table S10). GO and Reactome pathway analysis showed an enrichment of terms related to lipid metabolism and energy homeostasis (Fig. 4g). These genes code for several key enzymes of hepatic lipid metabolism including ATP-citrate lyase (*Acly*), Malic enzyme 1 (*Me1*) [67], acetoacetyl-CoA synthetase (*AACS2*) [68], pyruvate kinase (*PKLR*) [69], glycerol-3-phosphate acyltransferase (*GPAM*) [70], fatty acid elongase 6 (*Elovl6*) [71] and acetyl-CoA carboxylase 1 (*ACACA*) [72] (Fig. 4f). Notably, these genes are all direct targets of the transcription factors Srebf1 (also known as SREBP1) and ChREBP, two well-known regulators of hepatic lipogenesis [67, 73, 74]. Indeed, the *Srebf1* gene itself was hypermethylated and downregulated upon DR treatment (Fig. 4f). Hence, long-term DR-induced methylation and gene expression changes are specifically associated with hepatic lipid homeostasis.

**Fig. 5:**
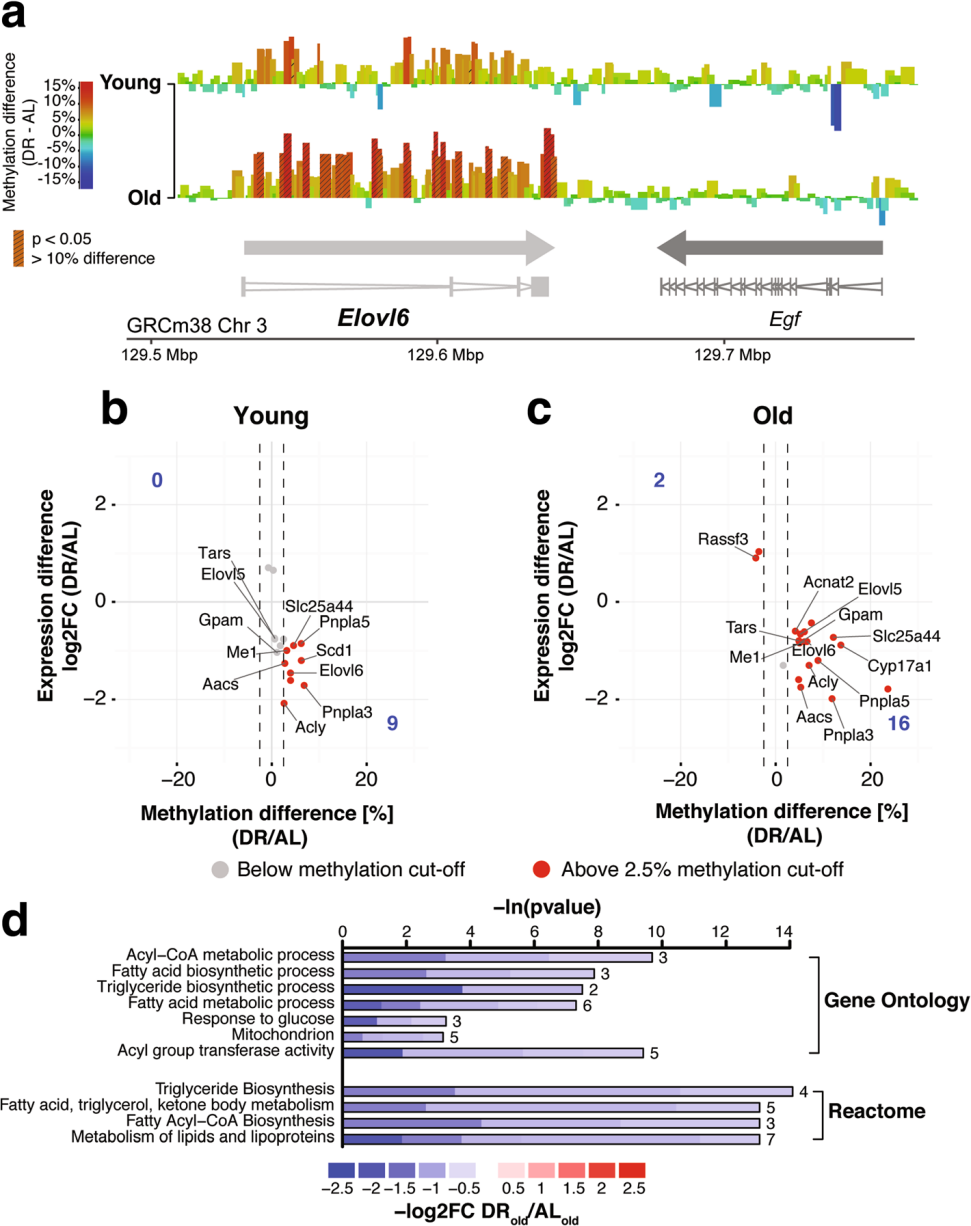
Dietary restriction (*DR*) caused differential whole gene body methylation of lipid metabolism genes. **a** Differential methylation landscape of the *Elovl6* gene locus at young and old age. Bins are represented as *bars* with color scale and height indicating methylation differences. *Hatched bars* indicate bins that show significantly different methylation between ad libitum (*AL*) and DR conditions. *Arrows* indicate gene orientation; merged mRNA structure is depicted below. **b**, **c** Scatterplot of differential expression versus whole gene body methylation differences at young (**b**) and old age (**c**). Number of genes per quadrant is denoted in corners; *dashed lines* indicate methylation difference cutoff of > ±2.5%. Fisher’s exact test indicates a significant (*p* < 0.032) inverse relationship (Pearson correlation −0.39) at old age but not at young age. **d** Gene ontology and reactome enrichment of genes with an inverse relationship of whole gene body methylation and differential expression at old age. Lengths of bars represent negative log-transformed, adjusted *p* values for Fisher’s exact enrichment test. Cells indicate log_2_-fold changes (*log2FC*) between AL and DR per gene

Interestingly, in 18 of the 56 genes, we observed methylation differences, for example in the *Elovl6* gene, that stretched over both introns and exons in the entire gene body but not the promoter region or neighboring genes (Fig. 5a; Additional file 14: Table S13). Quantification of whole gene body methylation by examining single bins per gene showed that all 18 genes had a whole gene body methylation difference of at least 2.5% between AL and DR at old age (Fig. 5b, c). Genes with whole body methylation differences were enriched for fatty acid, triglyceride and ketone body metabolism-related GO terms (Fig. 5d).

Many of the genes found differentially methylated under DR in old animals were already differentially expressed upon DR in young animals (Figs 4e and 5b). DR-induced differential gene expression may therefore precede gene body methylation changes, which may serve as a long-term memory of the DR state.

While DR predominantly induced hypermethylation of gene bodies correlated with repression, some exceptions were also found. For example, Insulin receptor substrate 2 (*IRS2*) and *Rassf3* showed hypomethylation (Additional file 1: Figure S10B, C) associated with increased expression in old DR animals (Figs 4f and 5c). *IRS2* is a key regulator of hepatic insulin resistance and has been associated with lifespan in mice [75]. *Rassf3* is a tumor suppressor and its expression is downregulated in several human tumors, including liver, stomach, colon and lung cancers [76]. Furthermore, hypermethylation of *Rassf3* associated with reduced gene expression correlates with tumorigenesis in pituitary somatotroph adenoma [77]. The anti-tumorigenic effect of DR may therefore include methylation-dependent regulation of *Rassf3* activity.

### Long-term dietary restriction shifts the hepatic triglyceride pool toward shorter fatty acid chain lengths

Several key enzymes of hepatic lipid metabolism were hypermethylated and transcriptionally downregulated in old DR animals (Fig. 4f). These included glycerol-3-phosphate acyltransferase, which catalyzes the first step in glycerolipid biosynthesis [78], and ATP-citrate lyase, which is responsible for the synthesis of cytosolic acetyl-CoA [79], a key metabolite for the production of fatty acids. Furthermore, fatty acid elongase 5 (*Elovl5*) and *Elovl6*, which catalyze the initial and rate-limiting steps in fatty-acid elongation [78–80], were also hypermethylated and downregulated by long-term DR.

**Fig. 6:**
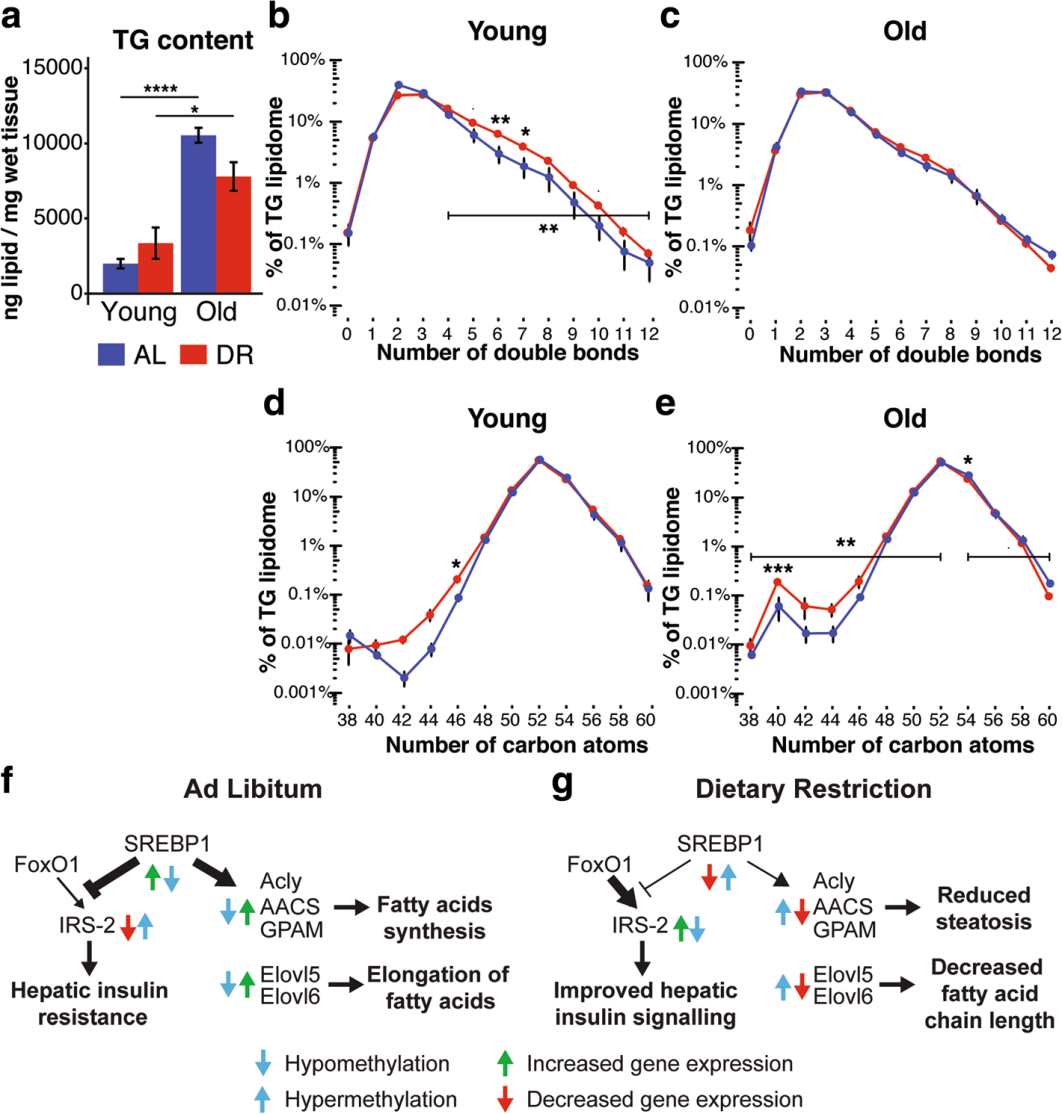
Lipidome profiling of dietary restriction (*DR*)-induced changes in triglyceride (*TG*) acyl chain length and saturation in the mouse liver. **a** Hepatic TG content of ad libitum-fed (*AL*) and DR animals at young and old age. There was a significant interaction between diet and age (two-way ANOVA *p* < 0.05). **b-e** Distribution of TG species in young (**b**, **d**) and old animals (**c**, **e**) classified according to the degree of saturation (**b**, **c**) and number of carbon atoms (**d**, **e**) as proxy for TG-associated fatty acid chain length. Values represent normalized relative abundances (0–100%) on a logarithmic scale. Indicated are *p* values for pairwise comparisons for each species (Tukey range test) and for specific intervals (paired Wilcoxon-rank-sum test). DR animals showed significantly more TGs with four or more double bonds at young age (b, *p* = 0.0039; paired Wilcoxon-rank-sum test) and significantly more TGs with 52 or fewer carbons at old age (e, *p* = 0.0078; paired Wilcoxon-rank-sum test). AL animals showed significantly more TGs with 54 or fewer carbons at old age (e, *p* = 0.052; paired Wilcoxon-rank-sum test). • *p* < 0.1, * *p* < 0.05, ** *p* < 0.01, *** *p* < 0.001. Error bars denote means ± SEM. **f**, **g** Schematic outline of differential methylation and gene expression in the Sreb1f (SREBP1) network under AL (**f**) and DR (**g**) conditions

Because DR downregulated the expression of genes involved in lipogenesis and fatty-acid elongation, we probed the functional significance of these changes by performing liquid chromatography–tandem mass spectrometry (LC-MS/MS) profiling of hepatic triglycerides (TGs) from young and old animals under DR and AL feeding. Total hepatic TG content was similar in young AL and DR animals, but showed a larger increase with age in AL animals, with a significant interaction between diet and age (Fig. 6a). DR thus ameliorated the age-related increase in hepatic TG levels. We assessed diet- and age-related TG composition by analyzing the saturation and chain length of TG-associated fatty acids (Fig. 6b–e). Surprisingly, relative saturation profiles differed only at young age, with DR animals having significantly more TGs with four or more double bonds (Fig. 6b), an effect not seen at old age (Fig. 6c). In contrast, DR induced a decrease in chain length of TG-associated fatty acids both in young and old animals (Fig. 6d, e), an effect that was already significant in young animals and became more prominent with age. In keeping with the observed decrease in fatty acid chain length in old DR animals, old AL animals showed elevated levels of TGs containing longer chain fatty acids. Hence, consistent with hypermethylation and downregulation of expression of key enzymes of hepatic lipid metabolism, in particular the elongases, DR protected the liver against age-related increase in TG content and induced a shift towards shorter chain length in TG-associated fatty acids and TGs.

## Discussion

DR extends lifespan and ameliorates many effects of aging on organismal and specific organ physiology, but its effects on the epigenome have not been systematically examined [81, 82]. This is important because dietary factors can alter epigenetic modifications and such alterations may have long-term consequences for gene expression and organ function [83]. Here we have profiled DNA methylation by whole genome BS-seq, the transcriptome and the lipidome in female mouse liver during aging with and without DR. Our key findings are that regions that become differentially methylated (DMRs) during aging, either by hypo- or hypermethylation, experience less change during DR. However, under DR there is a specific group of genes that acquire gene body methylation associated with their transcriptional repression, including key lipid regulatory genes. As a result, the age-associated increase in liver TG content is attenuated together with a reduction in TG acyl chain length. Hence DR generally protects from age-induced methylation changes and induces epigenetic reprogramming of lipid metabolism genes. Therefore, physiologically meaningful epigenetic changes occur during aging from which DR partially protects and simultaneously instigates a beneficial epigenotype.

We determined a small overall increase of CpG methylation in the liver during aging, consistent with identifying more hypermethylated than hypomethylated DMRs. Because there is generally both a loss and a gain of methylation during aging [84], the net effect on global methylation levels will likely depend on the tissue. Interestingly, under DR there was a concurrent reduction of the de novo methylase Dnmt3b and the dioxygenase Tet2. This would predict lower methylation turnover [84] and hence reduced erosion of methylation patterns over time, which is what we observed. Given that known signaling pathways regulate Dnmt3b (Erk1,2; Gsk3beta; [85]) and Tet2 (retinol and retinoic acid; [86]), the control of these pathways will be intricately integrated into regulated aging. Alteration of acyl chain length and saturation of membrane lipids will impact on the structure of the plasma and other membranes, which will lead to modification of membrane domains such as lipid rafts [87]. This in turn will modify the activation of PI3kinase and Raf signaling [88] upstream of Erk, GSKbeta and SREBP [89, 90]. Thus these lipid-modifying pathways, which modulate membrane fluidity, could be candidates for exploring for the signaling effects of DR.

Age-related DMRs were predominantly found in gene bodies, and age-related hypomethylated DMRs were associated with genes that regulate fatty acid, lipid and acyl-CoA metabolism, among others. Although globally there was no connection between methylation and transcription, which is not unexpected given the many different levels of epigenetic regulation, this signature is also one of the major ones at the transcriptional level, reflecting a consistent change in lipid metabolism during aging.

Age-related methylation changes will not only be influenced by the overall tuning of the methylation and demethylation machinery, but also by local interactions with chromatin and transcription factors [34, 65, 91]. Indeed we found that hypomethylated DMRs were generally enriched for open chromatin marks and transcription factor binding sites, while hypermethylated DMRs were more enriched for heterochromatic marks, reflecting established relationships with the DNA methylation machinery [34, 36, 91, 92]. At this point, however, this analysis is imperfect because histone mark profiling data are only available from young male animals; they may well change during aging or in a sex-specific manner, too. For example, hypermethylated age-related DMRs were enriched for repressive promoter chromatin characterized by the repressive mark H3K27me3. In contrast, DR-ameliorated DMRs, which no longer become hypermethylated during aging under DR, were not enriched for repressive promoter chromatin. This indicates the possibility that the H3K27me3 mark in these regions may become altered during aging. In our study we used liver samples from three female mice per treatment group, consistent with accepted guidelines for transcriptomics and DNA methylation studies [93–95]. Future work should extend our findings to male animals and other genotypes.

We identified a specific subset of 18 genes that showed DR-induced differential methylation spanning large parts of the gene body. DNA methylation in gene bodies has been proposed to function in the regulation of alternative splicing, silencing of alternative promoters or the regulation of other functional elements [96–98]. However, the differential whole gene body methylation detected here was relatively evenly distributed over the whole gene body and not associated with specific gene features like exons, introns or promoter elements, suggesting a different function. Whole gene body methylation might slow down transcription elongation rates [99, 100] and thereby contribute to the inverse relationship of gene expression and DNA methylation observed in these genes.

DR causes reduced hepatic lipogenesis and increased utilization of lipids as an energy source via enhanced lipolysis [101]. It thereby protects aged animals from steatosis and visceral fat accumulation [23], accompanied by the amelioration of age-dependent insulin resistance [101]. The transcription factor Srebf1 (SREBP1) is a key regulator of lipogenic enzyme expression in the liver [67, 73, 74] and mediates hepatic insulin resistance through inhibition of IRS-2 [102]. Interestingly, we found that the *Srebf1* gene itself and key Srebf1 target genes like *Acly*, *Me1* [67], *AACS* [68], *ACACA* [72], *GPAM* [70], and *Elovl5* [103] and *Elovl6* [71], which catalyze rate-limiting steps in fatty acid synthesis and fatty acid elongation [104], respectively, were hypermethylated and transcriptionally downregulated in response to DR (Fig. 6f, g). Consistent with our data, downregulation of *Srebf1* has been suggested to play an important role in gene regulation upon DR in rodents [18]. Furthermore, DR caused hypomethylation of the *IRS-2* gene and increased *IRS-2* expression. *IRS-2* is a direct Srebf1 target gene and is upregulated during nutrient deprivation in the liver via the Foxo1 transcription factor. Under AL-fed conditions, Srebf1 interferes with the binding of Foxo1 to the *IRS-2* promoter and thereby represses *IRS-2* expression [102]. Loss of IRS-2 function results in hepatic insulin resistance [105], an important pathophysiological feature of age-related type 2 diabetes. In summary, our data suggest epigenetic regulation of the Srebf1 network as an integral mechanism by which DR protects organisms against age-related steatosis and hepatic insulin resistance.

Consistent with hypermethylation and transcriptional downregulation of fatty acid elongases under DR, we observed a shift in the hepatic triglyceride pool toward medium-chain triglycerides (MCTs). Dietary supplementation of MCTs has been shown to prevent diet-induced liver pathologies and insulin resistance and is used in the treatment of refractory childhood epilepsy [106–108]. Interestingly, DR treatment has been shown to reduce seizures in rodent epilepsy models [109, 110]; however, whether MCTs are involved in the beneficial effects of DR is currently unknown and could be explored in future studies. The age-related shift in acyl chain length will additionally reduce the energy metabolic capability of the cells and can have further functional consequences, such as different fatty acid species being generated through ATGL-catalyzed TG hydrolysis, which could for example have distinct PPARα-activating capacity [111]. Further, an increase in saturated TGs will increase saturated diaglycerides that have been shown to decrease insulin sensitivity with a consequent promotion of obesity [112]. Conversely, the DR-induced change in lipid elongases and other enzymes will positively regulate insulin sensitivity, a key event in healthy aging. Notably this will parallel the increase in *IRS-2* expression, which will further increase insulin signaling, improving the metabolic health of animals during aging.

## Conclusions

This study provides novel insights into the effect of dietary restriction (DR) on age-related DNA methylation. DR caused remodeling of genome-wide patterns of DNA methylation so that age-related changes were delayed, while changes at specific loci involved in lipid metabolism affected gene expression and the resulting lipid profile. Our work has therefore uncovered an epigenetic axis that links the long-term beneficial effects of DR with specific transcriptional and functional outcomes on metabolism.

## Methods

### Animals and DR protocol

The DR study was performed in accordance with the recommendations and guideline of the Federation of the European Laboratory Animal Science Association (FELASA), with all protocols approved by the Landesamt für Natur, Umwelt und Verbraucherschutz, Nordrhein-Westfalen, Germany. Animals used in this study were female F1 hybrid mice (C3B6F1) generated by mating C3H/HeOuJ females with C57BL/6 N males. Parental animals were received from Charles River Laboratories (Lyon, France) and breeding of experimental animals was done in the in-house animal facility. Litter size was adjusted to a maximum of eight pups by culling surplus male pups within 3 days of birth and animals were weaned at 21–28 days of age. Animals were housed in groups of five in individually ventilated cages under specific-pathogen-free conditions with constant temperature (21 °C) and humidity (50–60%) and a 12-hour light/dark cycle. All mice were fed commercial rodent chow (ssniff R/M-H autoclavable, ssniff Spezialdiäten GmbH, Soest, Germany) and were provided with acidified water ad libitum. The food uptake of AL-fed animals was measured weekly and DR was applied by feeding DR animals 40% less food. Adult-onset DR treatment was started at the age of 12 weeks in a stepwise manner, by reducing food amounts fed to DR animals by 10% each week until the 40% reduction was reached. DR animals were fed once a day and all animals were checked daily for their well being and any deaths.

### Tissue sample collection

At the age of 5 and 27 months, which corresponded to 2 and 24 months of DR treatment, respectively, mice were killed by cervical dislocation and organs were immediately harvested and flash-frozen. On the day they were killed, tissue samples were collected in a 3 h time-window in the morning prior to the regular feeding of DR mice.

### RNA-seq measurement and analysis

RNA from three AL and three DR animals per time point was isolated from 30 mg of liver tissue using Trizol Reagent (Thermo Fisher Scientific, Darmstadt, Germany) according to the manufacturer’s instructions, followed by DNAse treatment with the TURBO DNA-free Kit (Thermo Fisher Scientific). RNA integrity was analyzed using the Agilent TapeStation System (Agilent Technologies, Frankfurt, Germany). RNA-seq library preparation and sequencing was performed by the Max Planck-Genome-centre Cologne, Germany (http://mpgc.mpipz.mpg.de/home/). Stranded TruSeq RNA-seq library preparation was conducted as previously described [113] using 3 μg of total RNA as input and rRNA depletion. Barcoded libraries were sequenced with 2 × 40 mio, 100 bp paired-end reads on an Illumina HiSeq2500 (Illumina, San Diego, California, USA). Raw sequence reads were trimmed to remove adaptor contamination and poor-quality reads using Trim Galore! (v0.3.7, parameters: --paired --length 25). A modified mouse reference genome (build GRCm38), with all known single nucleotide polymorphisms replaced by ‘N’ was set up to account for the hybrid genome of the C3B6F1 mice. Trimmed sequences were aligned using Tophat2 [114] (v2.0.14, parameters: --no-mixed --library-type = fr-firststrand -g 2 -p 15 -r 500 --mate-std-dev 525), supplying GENCODE annotation [115] (release M9, main annotation) for improved mapping. Multi-mapped reads were filtered using samtools [116] (v1.2, parameters: view -F 0x100 -b –h). Data visualization and analysis was performed using SeqMonk, ClueGO [117] custom RStudio and the following Bioconductor packages: Deseq2 [118], topGO [119], org.Mm.eg.db. For Figs. 4g and 5d, we further used the CellPlot package (https://github.com/dieterich-lab/CellPlot). To account for tissue-specific expression, we defined all genes passing the independent filtering of Deseq2 [118] as “expressed” (13,649 genes in total). DEGs were determined using Deseq2’s Wald test [118] run across all four treatment groups (age and diet), with subsequent pair-wise contrasts. *p* values were adjusted for multiple testing. Genes were considered to be significantly differentially expressed with a an adjusted *p* value <0.05 and no cutoff for fold change was used. Unless stated otherwise, the set of expressed genes was used as background for all functional enrichment analyses involving expression data.

### cDNA synthesis and qRT-PCR

For qRT-PCR validation of gene expression changes, RNA was isolated from an independent set of three AL and three DR animals per time point. RNA isolation was done as described for the RNA-seq experiment. cDNA was synthesized from 5 μg of RNA using SuperScript VILO Master Mix (Thermo Fisher Scientific) according to the manufacturer’s instructions. qRT-PCR was performed using TaqMan Gene Expression Assays (Thermo Fisher Scientific) in combination with TaqMan Gene Expression Master Mix according to the manufacturer’s instructions. Liquid handling was done with a Janus Automated Workstation (PerkinElmer, Waltham, Massachusetts, USA) and cycling was carried out on a 7900HT Fast Real-Time PCR System (Thermo Fisher Scientific). Relative expression was calculated using the ΔΔC_T_ method and Polr2i as normalization control. The following TaqMan gene expression assays were used: *Pnpla3* (Mm00504420_m1), *Fasn* (Mm00662319_m1), *Irs2* (Mm03038438_m1), *Cyp3a11* (Mm00731567_m1), *Tet3* (Mm00805756_m1), *Gna14* (Mm00492374_m1), *GNMT* (Mm00494688_m1) and *Polr2i* (Mm01176661_g1).

### BS-seq measurement and differential methylation analysis

DNA of three AL and three DR animals per time point was isolated from 30 mg of liver tissue using the AllPrep DNA/RNA Mini Kit (Qiagen, Hilden, Germany) according to the manufacturer’s instructions. Around 400 ng of genomic DNA was used as input for BS-Seq library generation. DNA was sonicated, and end repair and A-tailing were performed using the NEBNext kit according to the manufacturer’s instructions (New England Biolabs, Frankfurt, Germany). Illumina’s Early Access Methylation Adaptor Oligo Kit was used for adaptor ligation. As described previously [120], adaptor-ligated DNA was treated with sodium-bisulfite using the Imprint DNA Modification Kit (Sigma-Aldrich, Munich, Germany) according to the manufacturer’s instructions for the one-step protocol. Bisulfite-treated DNA was amplified using PfuTurbo Cx Hotstart DNA Polymerase from Agilent Technologies with 14–18 cycles depending on input amount. Size selection was performed by gel extraction for DNA fragments between 200 bp and 250 bp. Libraries of young samples were sequenced using the paired-end protocol. Old sample libraries were sequenced using a single-end protocol and a subsequent re-run using a paired-end protocol to increase coverage. The data have been deposited in NCBI’s Gene Expression Omnibus [121] and are accessible through [GEO: GSE92486].

Raw sequence reads were trimmed using Trim Galore! (v0.4.2). Trimmed sequences were aligned using Bismark [122] (v0.16.3). Methylation calls were extracted after duplicate sequences had been excluded. Data visualization and analysis were performed using SeqMonk and custom RStudio scripts. Data from replicates of the same condition were merged using SeqMonk’s data group option, in order to enhance coverage and detection of subtle differences. However, we verified that the regions we identified as differentially methylated with age or DR were consistently regulated among replicates and not a result of strong methylation differences in single samples (Additional file 1: Figure S11A–D). Regions with an unusually high number of observations were detected and filtered using non-overlapping 25 kb windows, followed by read count quantification and subsequent BoxWhisker filter implemented in SeqMonk with stringency >10. To try to achieve a fair and unbiased analysis of the methylome, we constructed windows containing 50 CpGs over the whole genome, spaced 25 CpGs apart. Each window therefore contained around the same amount of data and all windows had similar technical noise and statistical power. Furthermore, to reduce the effect of coverage differences between samples, only cytosines covered by at least three observations in all conditions were used in the differential methylation analysis. Methylation for each window was calculated as the average of methylation for each covered CpG position. Windows that contained significantly different methylation levels (pairwise Chi-squared tests with subsequent multiple testing correction; adjusted *p* value <0.05) and a minimal difference cutoff of 10% were defined as differentially methylated (DMR). Compter (http://www.bioinformatics.babraham.ac.uk/projects/compter/) analysis was used to show that DMRs had no composition bias in their underlying sequence. Hypo- and hypermethylated DMRs showed the same sequence composition (data not shown). We defined three sets of DMRs: (1) age-related DMRs: DMRs showing a significant change under AL with age, that is, AL_young_ versus AL_old_; (2) Young-DR DMRs: showing significant change between AL_young_ and DR_young_; and (3) Old-DR DMRs: showing significant change between AL_old_ and DR_old_. For the example in Additional file 1: Figure S6A, the methylation landscape over bivalent CGI of *Nol3* was visualized by calculating average CpG methylation levels of 500 bp 250 bp overlapping bins to improve resolution.

### Profiling of ameliorated age-related methylation changes under DR

For each bin, we calculated differences between the diets after aging occurred, that is, at old age (DR_old_ versus AL_old_), and plotted these against differences during aging under either AL or DR conditions (AL_old_ versus AL_young_; DR_old_ versus DR_young_). We next performed linear regression and calculated the Pearson correlation coefficient for differences at old age between diets as a function of age-related differences under AL or DR. The observed slope of the linear fits and correlation coefficients were compared to permutation studies to confirm the robustness of our findings. Therefore, we permutated the treatment labels per bin and calculated correlation and slope as before, which resembles a relationship based on noise only. We repeated the permutations, randomizing treatment labels for AL_young_ with AL_old_ and DR_young_ with DR_old_ (“across age, within same diet”) to exclude general effects between the diets. Finally, we also randomized AL_young_ with DR_young_ and AL_old_ with DR_old_ (“across diets, within same age”), thereby averaging out differences between diets, focusing on general differences between young and old.

We next analyzed age-related DMRs scattering strongly from the linear fit under DR conditions (Fig. 3a, b), because these represent age-related effects ameliorated by DR. We used two standard deviations (2*σ; corresponding to ±9.68% methylation difference) of the distribution of residuals for aging under AL conditions as an estimate of naturally random scattering. We then determined which age-related DMRs scattered beyond this cutoff from the linear fit for aging under DR conditions. These were defined as DR-ameliorated age-related DMRs.

### Enrichment analyses of DMRs over genomic and epigenetic elements

For a given genomic element, we calculated the enrichment for a set of DMRs by counting the number of DMRs overlapping the element of interest and compared it to the set of background bins (whole genome; *n* = 1,167,959). One-sided Fisher’s exact test with subsequent multiple testing correction (if enrichment analysis was run over multiple elements) was performed to determine the statistical significance of enrichment. Because most elements showed highly significant enrichment, we –log-transformed the adjusted *p* values. The ratio of the observed DMR frequency and the average frequency across the genome (“observed/expected”) for individual elements were used to compare enrichments across elements and between DMR sets. This way we were able compare the strengths of enrichments across elements in the same analysis.

### Functional enrichment analyses of differentially methylated genes

Unless stated otherwise, we performed functional enrichment of genes overlapped by a defined number of DMRs using topGO [119] with all genes in the genome as background.

### coChIP-seq analysis

coChIP allows the co-occurrence of two histone modifications on the same nucleosome to be detected. We therefore retrieved previously published coChIP-seq data [61] from the Gene Expression Omnibus repository and re-analyzed them. The analyzed data measured coChIP against H3K4me3-H3K27me3 in 8–12-week-old female C57BL/6 J mice in two biological replicates. Raw sequence reads were trimmed to remove adaptor contamination and poor-quality reads using Trim Galore! (v0.3.7, parameters: --paired --length 20). Trimmed sequences were aligned using Bowtie2 [123] (v2.0.14, default parameters). The resulting files were merged and multi-mapped reads discarded using samtools [116] (v1.2, parameters: view -F 0x100 -b –h). Peak calling was performed using MACS2 [124] (v2.1.1, parameters: callpeak -g hs –n test –B –q 0.01). Data visualization and mapping to CGIs was performed using SeqMonk and custom RStudio.

### Identification of whole gene body methylation

To identify genes that showed consistent differential methylation across their gene body, we tested each gene for significant enrichment of overlapping DMRs compared to all bins overlapping. A minimum of four overlapping bins was required. *p* values from Fisher’s exact test were corrected for multiple testing (Benjamini-Hochberg). Methylation of genes with a *p* value <0.05 was quantitated using all CpGs within the gene body and a minimal difference of ±2.5% was set as cutoff.

### Correlation analysis of methylation and transcription

To test for correlation between differential methylation and gene expression, we considered only genes overlapped by at least two DMRs. We averaged methylation differences for multiple DMRs overlapping the same gene before plotting, and only retained those showing at least 10% average difference. We thereby avoided unclear cases with an equal extent of hyper- and hypomethylation occurring over the same gene. We plotted log_2_-fold expression changes versus methylation differences, and the distribution of genes among the four resulting quadrants was tested for directionality using Fisher’s exact test. We additionally calculated a Pearson correlation estimate for all genes above the 10% (2.5% for whole body gene methylation) methylation cutoff. Genes that showed a significant correlation of methylation and gene expression were analyzed for functional enrichment using topGO [119].

### Triglyceride lipidome measurement and analysis

Extraction, measurement and quantification of lipids was performed as previously described [125], with 800 ng of synthetic 17:1/17:1/17:1-TG standard. We analyzed liver tissue from four young AL and four young DR mice, as well as three old AL and three old DR mice. We analyzed TG content using two-way ANOVA to test for an interaction between the factors age (young, old) and diet (AL, DR). Individual lipid species were calculated as the percent of the entire TG lipidome. For elongation and saturation analysis, TG species with the same number of carbons/double bonds were calculated as a percentage of the entire TG lipidome. We conducted one-way ANOVA with post-hoc Tukey HSD for each chain length/saturation to test for significant differences. In order to test whether the DR-induced shift towards shorter TG acyl chain composition was significant (Fig. 6), we averaged replicates of the same condition for each chain length and ran a pairwise Wilcoxon rank-sum test across the left (38:x to 52:x) and right tail of the chain length plot (54:x to 60:x). A saturation analysis was run accordingly, analyzing the interval from 0–3 and 4–12 double bonds.

### Definition of genomic and epigenetic elements

Gene annotation used in this study was obtained from the UCSC Genome Browser database [126]. We defined further genome annotation as follows:

**Promoter region:** Manually defined region stretching from 5 kbp upstream and 100 bp downstream of the transcription start site.

**CGI:** Annotation based on CXXC affinity purification plus deep sequencing (CAP-seq) experiments [127]. CGIs were further classified unambiguously into promoter, gene and intergenic CGIs. If a CGI overlapped both promoter and gene body, it was classified as a promoter CGI.

**Repetitive elements:** Annotation based on Repbase [128] and obtained via the UCSC Table Browser [129].

**Histone modification ChIP-seq peaks:** Publicly available, pre-processed H3K9ac, H3K4me1, H3K27ac, H3K4me3, H3K79me2, H3K27me3 and H3K36 ChIP-seq peaks from liver tissue of 8-week-old male C57BL/6 mice were obtained from the ENCODE dataset [57] and re-mapped to the GRCm38 genome using UCSC Genome Browser’s LiftOver [130].

**Active enhancer:** H3K4me1 mark overlapping the H3K27ac mark; further classified into geneic (overlapping gene or promoter) and distal.

**Active promoter chromatin:** H3K4me3 mark overlapping the H3K9 mark within 100 bp, without overlapping the H3K27me3 mark within 100 bp distance. Further overlapping the promoter region.

**Repressive promoter chromatin:** Narrow H3K27me3 mark (smaller than 3.5 kbp) that did not overlap H3K27ac or H3K4me3 within 100 bp distance. Overlapping the promoter region.

**Repressive geneic chromatin:** H3K27me3 mark that did not overlap H3K27ac or H3K4me3 within 100 bp distance. Overlapping the gene body. Where the repressive mark overlapped promoter and gene, it was classified as a promoter mark.

**Bivalent CGI:** CGI overlapped by one or more coChIP-seq peaks (against H3K4me3-H3K27me3). We did not differentiate between promoter or gene body CGIs. For annotation and enrichment analyses, bivalent CGIs were also counted when a DMR mapped to the CGI but did not overlap the H3K4me3-H3K27me3 peak.

## Additional files

Additional file 1: Supplementary Figures S1–11. (DOCX 12605 kb)

Additional file 2: Table S1. Functional enrichment and clustering results for differentially expressed genes. (XLS 140 kb)

Additional file 3: Table S2. Genes associated with age-related hypomethylation. (XLSX 284 kb)

Additional file 4: Table S3. Genes associated with age-related hypermethylation. (XLSX 396 kb)

Additional file 5: Table S4. Functional enrichment for genes associated with age-related hypermethylation. (XLSX 94 kb)

Additional file 6: Table S5. Functional enrichment for genes associated with age-related hypomethylation. (XLSX 64 kb)

Additional file 7: Table S6. Functional enrichment for genes associated with DR-ameliorated, age-related hypermethylation. (XLSX 57 kb)

Additional file 8: Table S7. Functional enrichment for genes associated with DR-ameliorated, age-related hypomethylation. (XLSX 38 kb)

Additional file 9: Table S8. Bivalent CGIs undergoing age-related differential methylation. (XLSX 54 kb)

Additional file 10: Table S9. Genes associated with Young-DR DMRs. (XLSX 387 kb)

Additional file 11: Table S10. Genes associated with Old-DR DMRs. (XLSX 249 kb)

Additional file 12: Table S11. Functional enrichment of genes associated with Old-DR-related hypermethylation. (XLSX 62 kb)

Additional file 13: Table S12. Functional enrichment of genes associated with Old DR-related hypomethylation. (XLSX 93 kb)

Additional file 14: Table S13. Genes with DR-induced whole gene body methylation differences. (XLSX 57 kb)
