# Supplementary material for: Dietary restriction protects from age-associated DNA methylation and induces epigenetic reprogramming of lipid metabolism
Source: Genome Biol. 2017 Mar 28;18:56. doi: 10.1186/s13059-017-1187-1 (PMC5370449; doi:10.1186/s13059-017-1187-1)
Supplement: Supplementary file 1 — Supplementary Figures S1–11. (DOCX 12605 kb) [file 13059_2017_1187_MOESM1_ESM.docx]

**
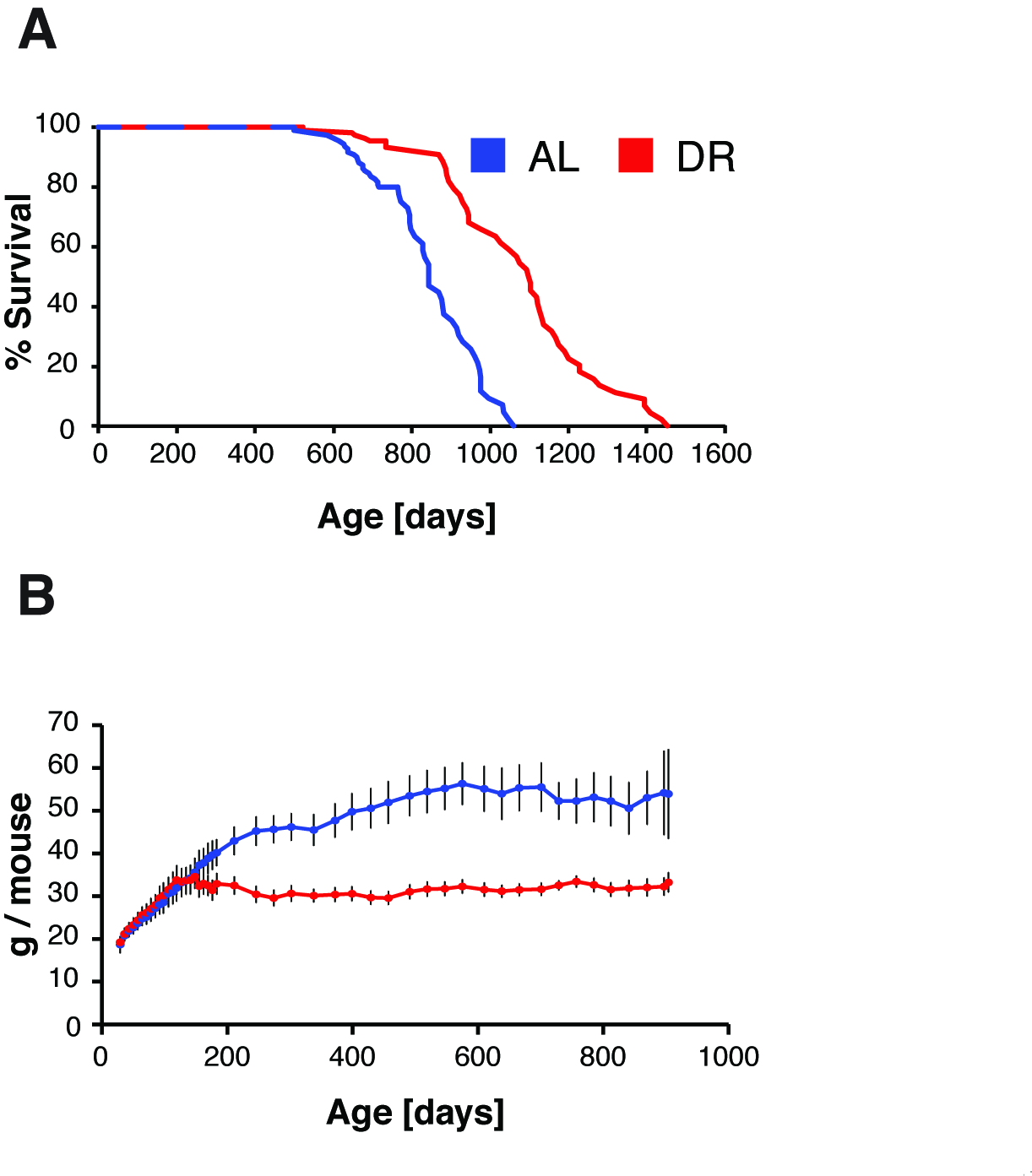
**

**Additional file 1: Fig. S1. DR treatment extended lifespan and reduced body weight of C3Bl6F1 hybrid female mice.** (A) Survival curves of AL and DR animals. Median lifespan of the AL- and DR-fed cohort was 843 and 1098 days, respectively, which corresponded to a 30% lifespan extension under DR treatment. (B) Body weight of AL and DR animals throughout life.

**
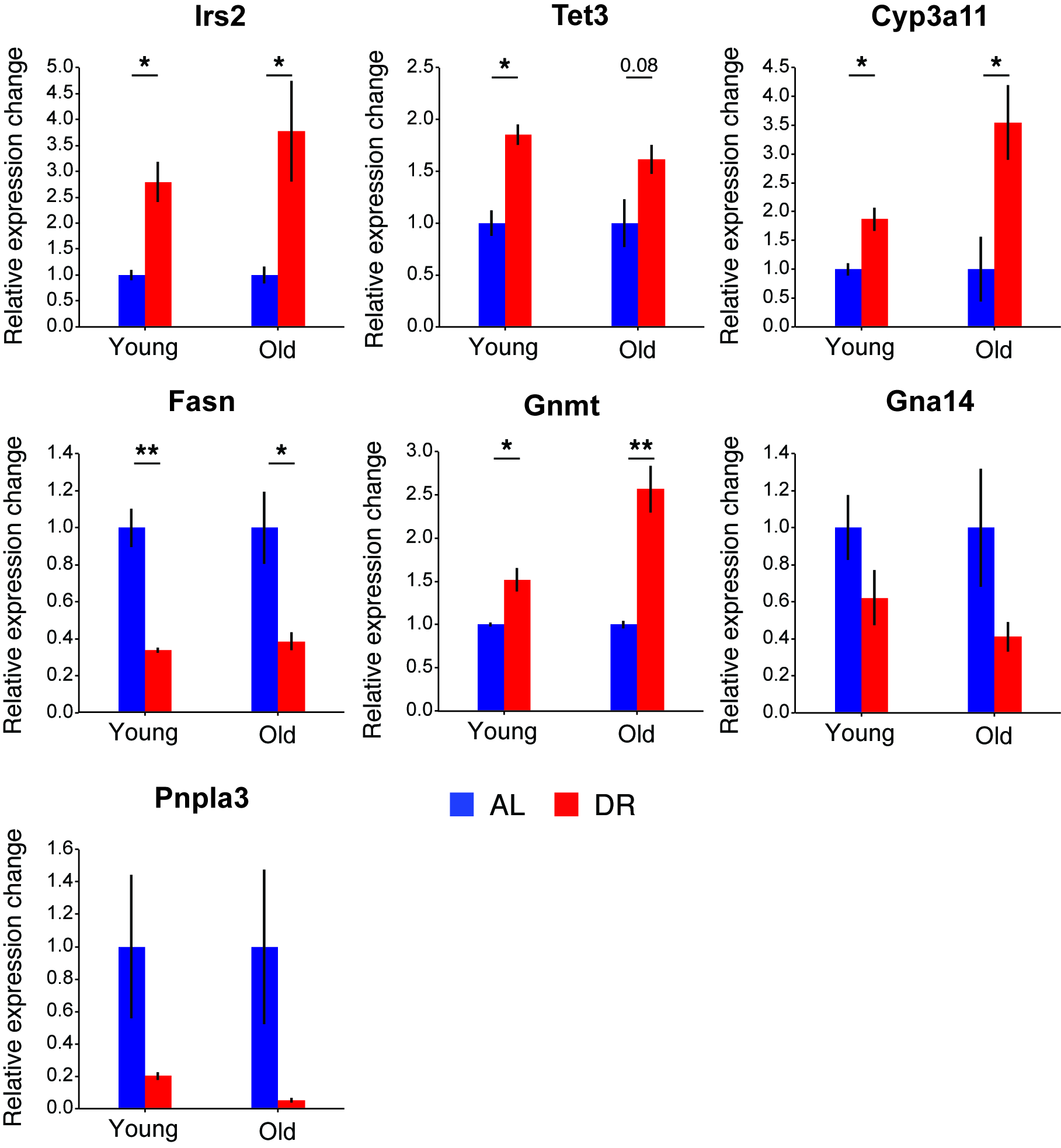
**

**Additional file 1: Fig. S2. Validation of differential expression of candidate genes by Q-RT-PCR**  Expression levels of selected genes as measured by Q-RT-PCR in three independent mouse liver samples per treatment. The Q-RT-PCR results are consistent with the gene expression changes observed in the RNA-seq analysis. *** p<0.001, ** p<0.01, * p<0.05, *Student’s t* test.

**
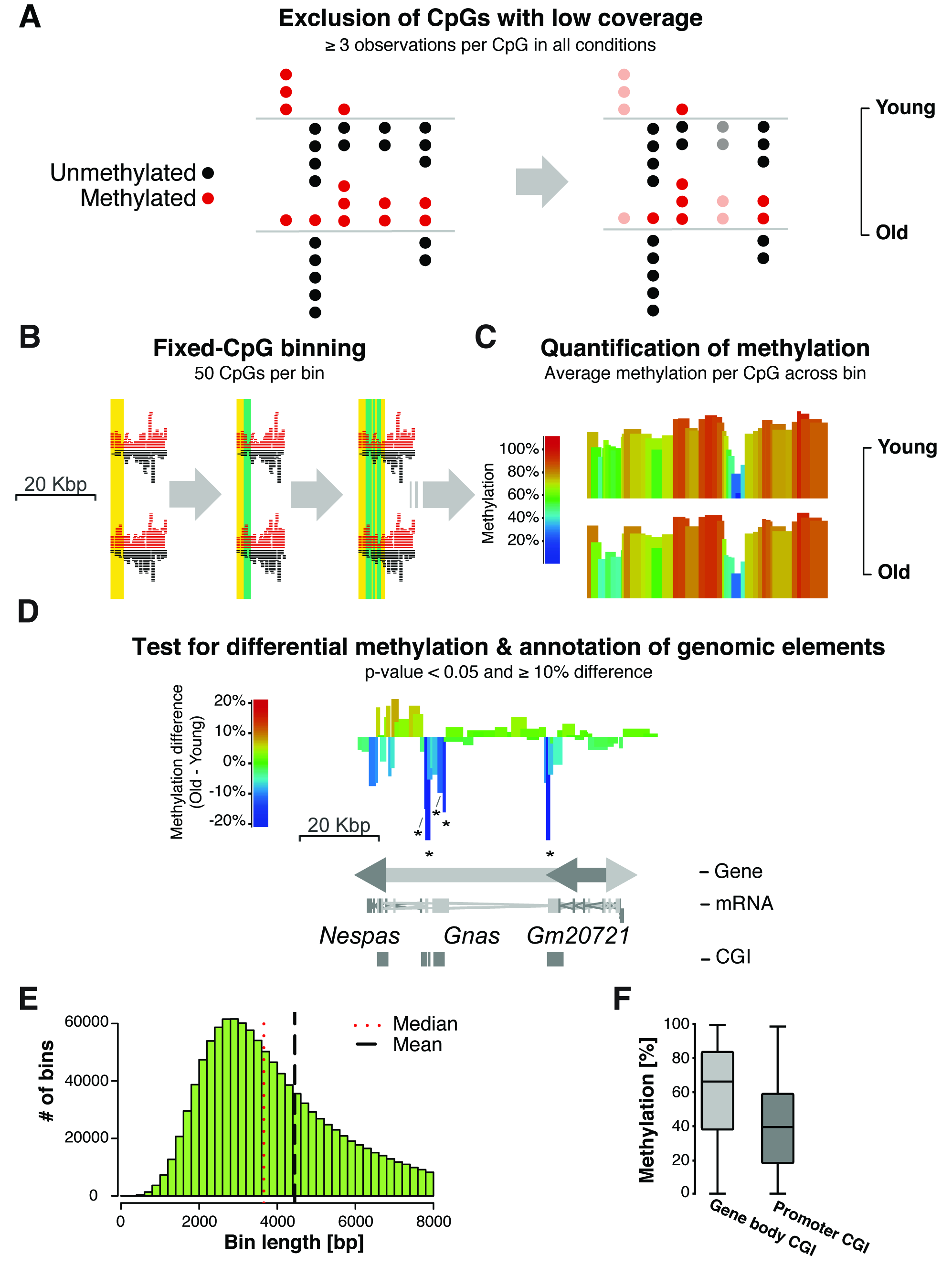
**

**Additional file 1: Fig. S3. Schematic outline of the method employed to quantify DNA methylation differences.** (A) Exclusion of CpGs with low coverage or inter-treatment different coverage. In the first analysis step all CpGs with less than three observations in each treatment group were filtered out (excluded CpGs are indicated by faded dots in the example). (B) 50 CpGs each were binned into one observation window (yellow). Windows overlapped by 25 CpGs. The sliding binning was conducted throughout the sequenced part of the genome. Widths of bins in bp varies with local CpG density. (C) Quantification of bin-wise methylation levels. For each 50 CpG window average methylation levels were calculated. Values range from 0%, indicating the completely demethylated state to 100% indicating complete methylation of all CpGs. For graphical representation methylation levels were indicated by height and color of bins. (D) In the next step significantly differentially methylated bins were identified by Chi square test using an adjusted p-value < 0.05 as cut-off. Furthermore, only bins with at least 10% methylation difference between treatment groups were considered for further analysis. For visualization, methylation differences were calculated by subtracting bin-wise methylation values between treatments. Color and height of bars represent methylation differences. Annotation of significant bins overlapping with genomic elements, such as genes, exons and introns or CGIs was performed. (E) Histogram of bin lengths (n = 1167959). For illustrative purpose, the histogram was calculated for > 90% of all bins. Mean and median bin lengths were 4450 bp and 3657 bp, respectively. (F) Boxplot representation of methylation values of all bins overlapping promoter or geneic CGIs.

**
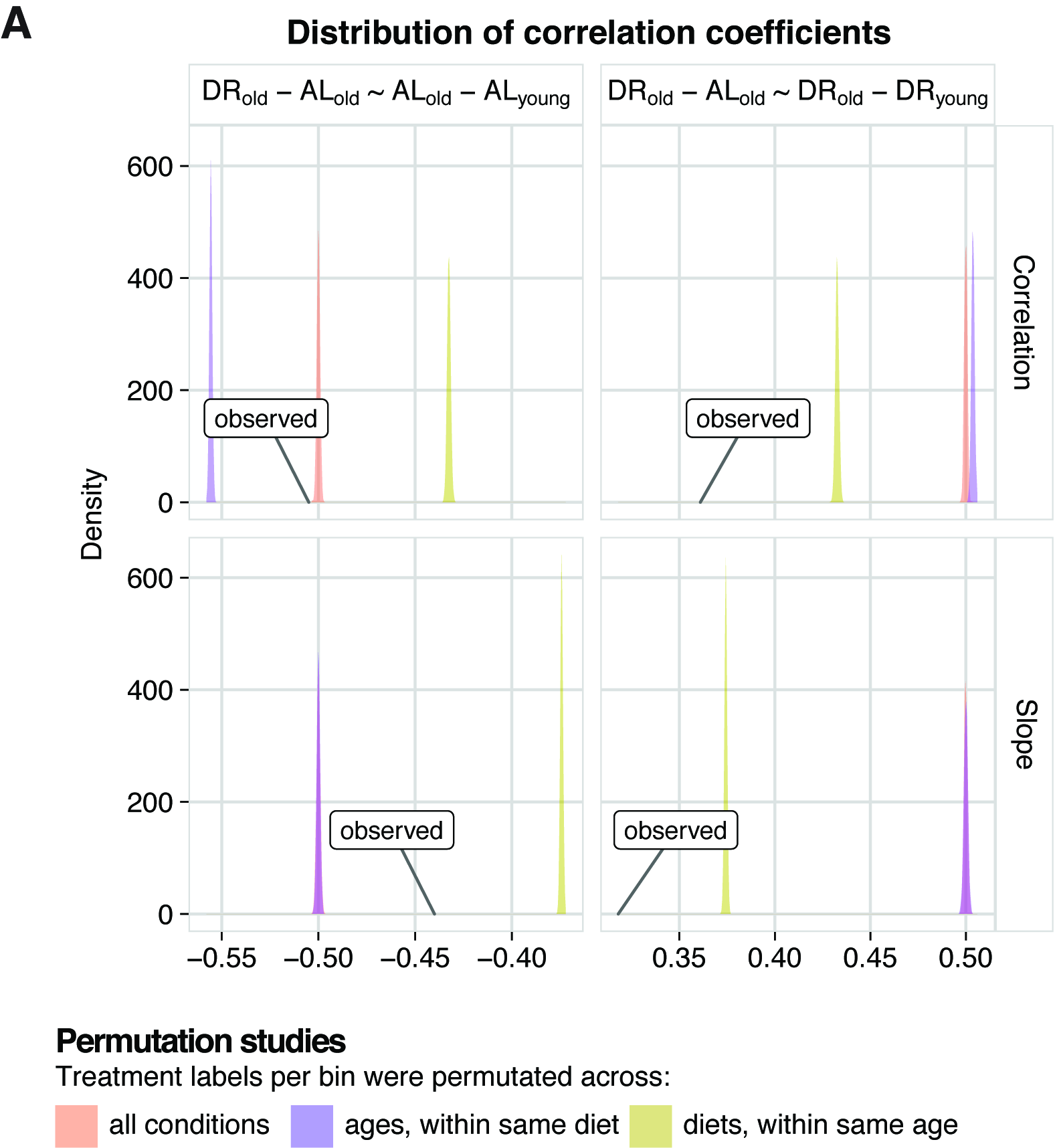
**

**Additional file 1: Fig. S4. Permutation analysis showed that the global amelioration of age-related DMRs by DR is not an artifact cause by noise in the dataset.** (A) Distributions of correlation and slope as computed by 1000 bin-wise permutations across all four treatment groups, across age of the same diet (AL_young_ and AL_old_ as well as DR_young_ and DR_old_; purple) and across diet groups of the same age (DR_young_ and AL_young_ as well as DR_old_ and AL_old_, yellow). Correlation and slope were calculated for comparing bin-wise differences between diets at old age (DR_old_ – AL_old_) with age-related changes under AL (AL_old_ – AL_young_) or DR feeding (DR_old_ - DR_young_). Values for experimentally observed data are indicated.

**
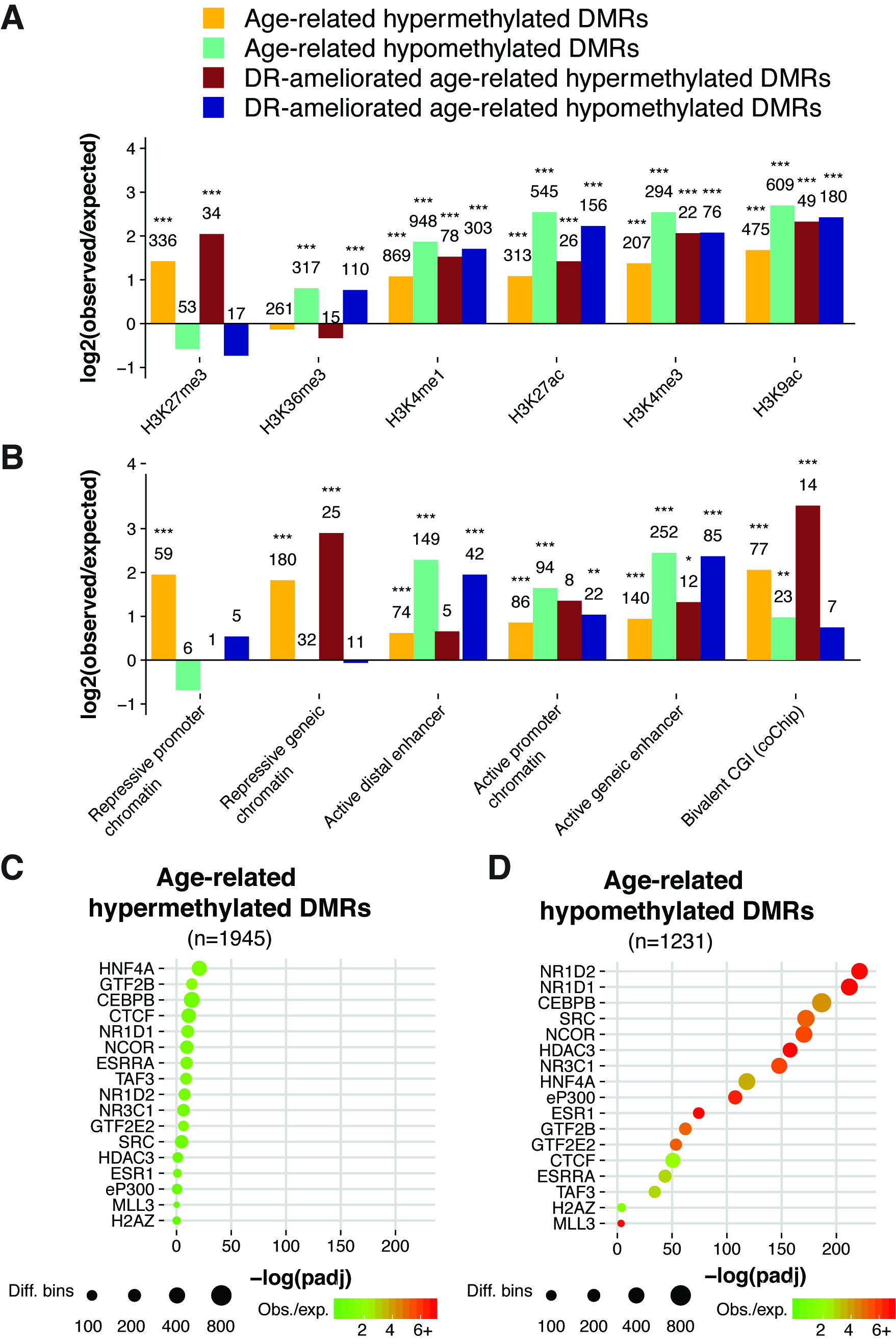
**

**Additional file 1: Fig. S5. Enrichment analysis of age-related DNA methylation changes.** (A) Enrichment analysis over histone modification ChIP-seq peaks and (B) over regulatory elements. Bars indicate the ratio of the observed age-related DMR frequency and the average frequency across the genome for age-related hypo- (n = 1231) and hypermethylated (n = 1945) and DR ameliorated hypo- (n = 439) and hypermethylated (n = 128) DMRs. (B) Enrichment analysis of age-related methylation over regulatory elements based on preprocessed ENCODE ChIP-seq and re-analyzed H3K4me3-H3K27me3 coChIP-seq data (For details for definition of regulatory elements see supplemental methods). Bars indicate the ratio of the observed DMR frequency and the average frequency across the genome for age-related hypo- (n = 1231) and hypermethylated (n = 1945) and for DR-ameliorated age-related hypo- (n = 439) and hypermethylated (n = 128) DMRs. (-log2-transformed p-values; *** p<0.001, ** p<0.01, * p<0.05, *Fisher’s exact* test) (C-D) Enrichment analysis of age-related hyper- (C) and hypomethylation (D) over DNA binding elements based on preprocessed ChIP-seq data obtained via the Cistrome Databrowser.

**
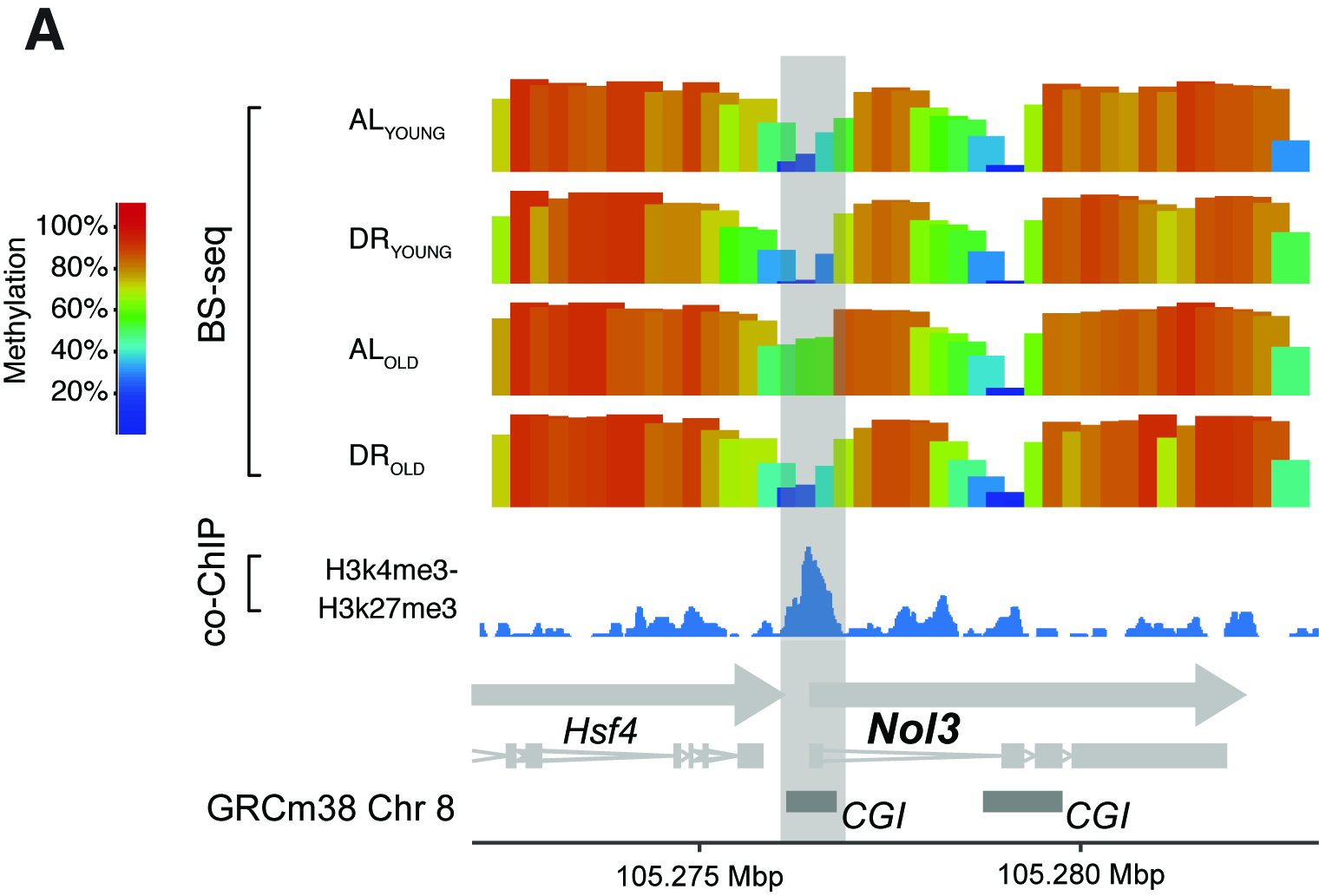
**

**Additional file 1: Fig. S6. DR-ameliorated age-related DNA methylation at the bivalent promoter of the Nol3 gene locus.** (A) Differential methylation landscape and H3K4me3-H3K27me3 coChIP-seq profile over bivalent CGI within the Nol3 promoter. (A) The shaded area indicates DR-ameliorated age-related DMRs. For improved resolution, the methylation profile is represented by 500 bp bins overlapping adjacent bins by 250 bp.

**
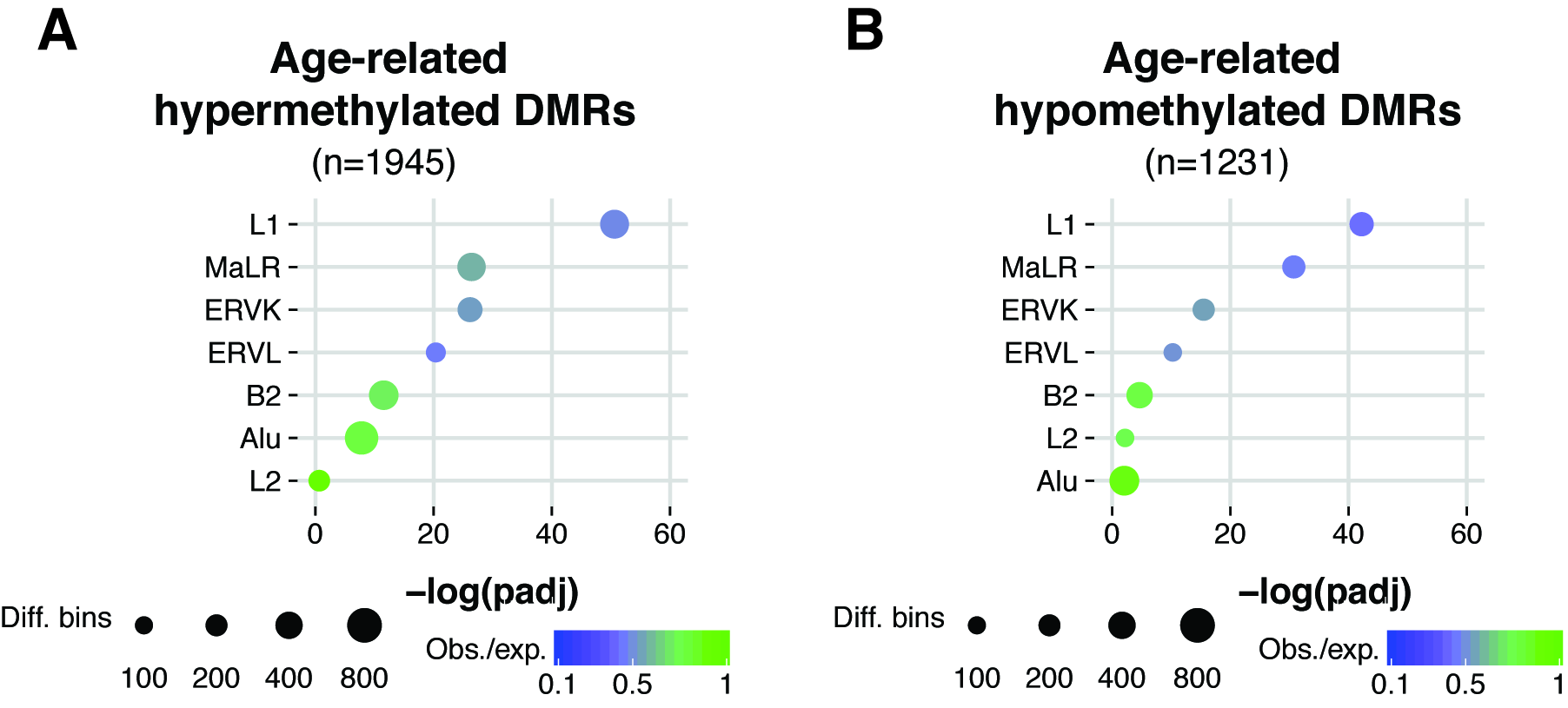
**

**Additional file 1: Fig. S7. Age-related DNA methylation changes were not enriched over repetitive elements.** (A-B) Enrichment analysis of age-related hypo- and hypermethylation over selected repetitive element families based on RepeatMasker data. X-axis indicates log-transformed p-values for one-sided *Fisher’s* exact test.

**
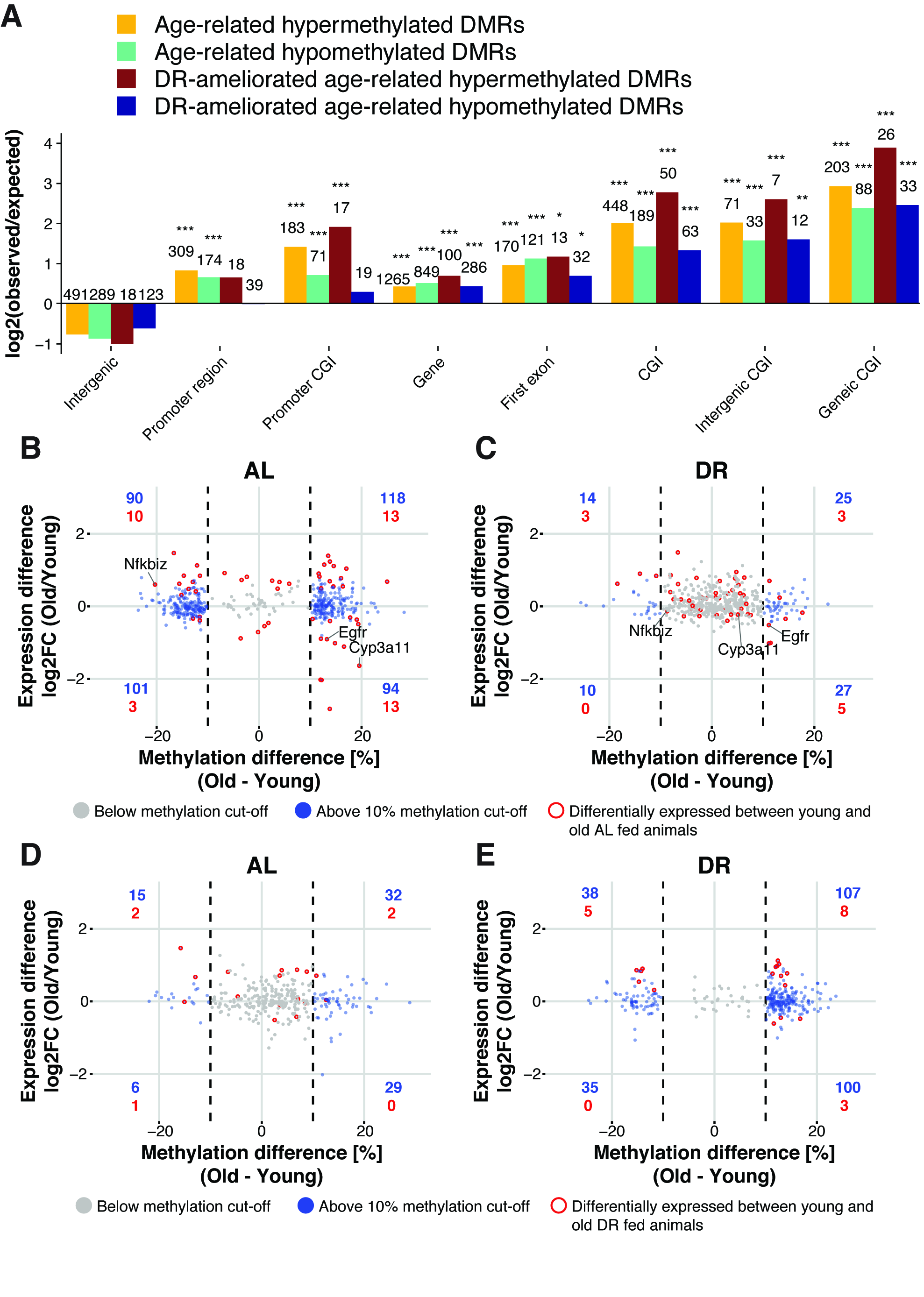
**

**Additional file 1: Fig. S8. Age-related effects on transcription and methylation do not correlate on a global scale.** (A) Enrichment analysis of age-related DNA methylation over genomic elements. Bars indicate the ratio of the observed DMR frequency and the average frequency across the genome (-log2-transformed p-values; *** p<0.001, ** p<0.01, * p<0.05, *Fisher’s exact* test) for age-related hypo- (n=1231) and hypermethylated (n=1945) DMRs and for DR-ameliorated hypo- (n=439) and hypermethylated (n=128) age-related DMRs. (B-C) Scatterplot of age-related differential methylation under AL versus gene expression under AL (B) and DR (C) feeding. There was no significant correlation between differential DNA methylation and gene expression (Fisher’s exact test, Pearson correlation) (D-E) Scatterplot of age-related differential methylation under DR conditions versus gene expression under AL (B) and DR (C) feeding. There was no significant correlation between differential DNA methylation and gene expression (Fisher’s exact test, Pearson correlation). Dashed lines indicate methylation cut-off of >±10%. Number of all genes and differentially expressed genes per quadrant are indicated in blue and red, respectively.

**
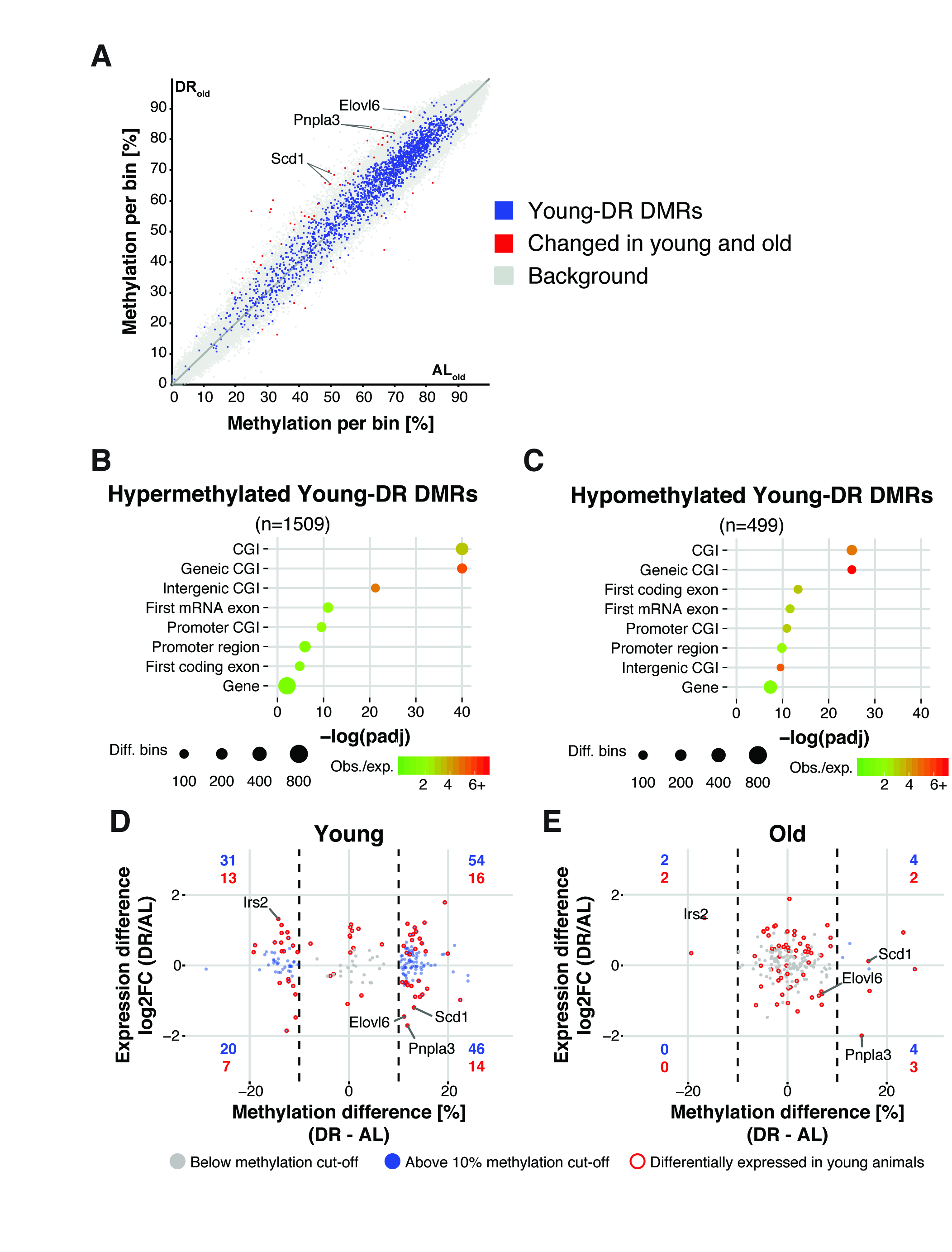
**

**Additional file 1: Fig. S9. DNA methylation changes induced by DR in young animals.** (A) Scatterplot representation of methylation values of each bin in old AL and DR animals. DR induced DNA methylation changes in young animals (Young-DR DMRs) are highlighted in blue. Bins that were significantly changed in young and old animals are highlighted in red. Bins that were not differentially regulated at any age (background) are represented in grey. (B-C) Enrichment analysis of hypermethylated (B) and hypomethylated (C) Young-DR- DMRs over genomic elements revealed only weak enrichment over genes or promoter elements. X-axis indicates log-transformed p-values for one-sided *Fisher’s* exact test. (D-E) Scatterplot of differential methylation upon DR in young animals versus gene expression in young (D) and old (E) mice. There was no significant correlation between differential DNA methylation and gene expression (Fisher’s exact test, Pearson correlation). Dashed lines indicate methylation cut-off of >±10%. Number of all genes and differentially expressed genes per quadrant are indicated in blue and red, respectively.

**
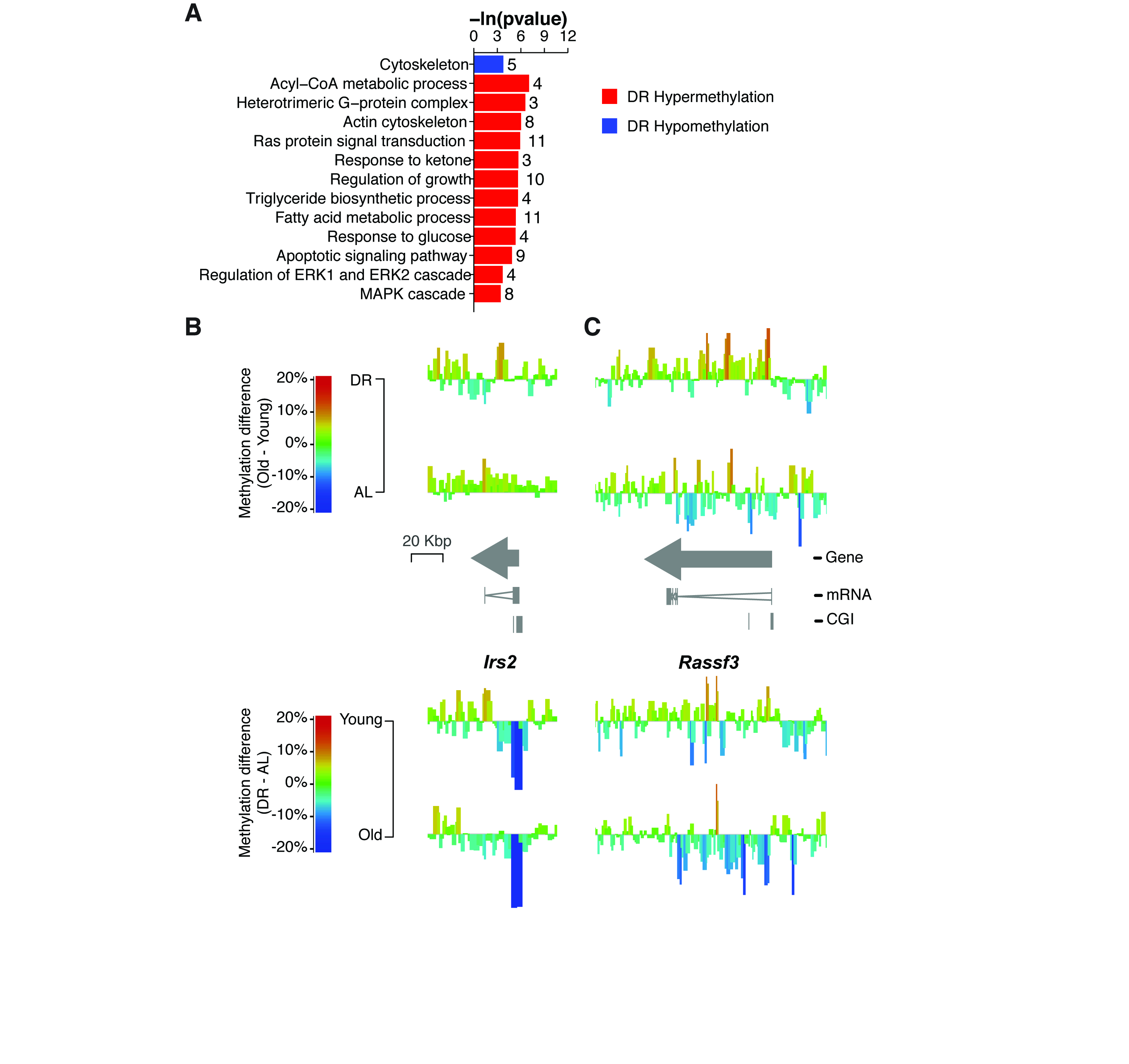
**

**Additional file 1: Fig. S10. Functional enrichment of genes differentially methylated by long-term DR.** (A) Functional enrichment of DR-induced differentially methylated genes in old animals. Enrichment analysis was performed for age-related hyper- and hypomethylation separately. Lengths of bars represent negative log-transformed, adjusted p-values for the Fisher’s exact enrichment test. Gene numbers associated with a given term are indicated. (B-C) Differential methylation landscapes of the Irs2 (B) and Rassf3 (C) genes. Both genes showed no differential methylation with age (upper panel) but were hypomethylated in response to DR (lower panel) and transcriptionally upregulated. Bins are represented as bars with color scale and height indicating methylation differences. Arrows indicate gene orientation; merged mRNA structure and location of CGIs are depicted below.

**
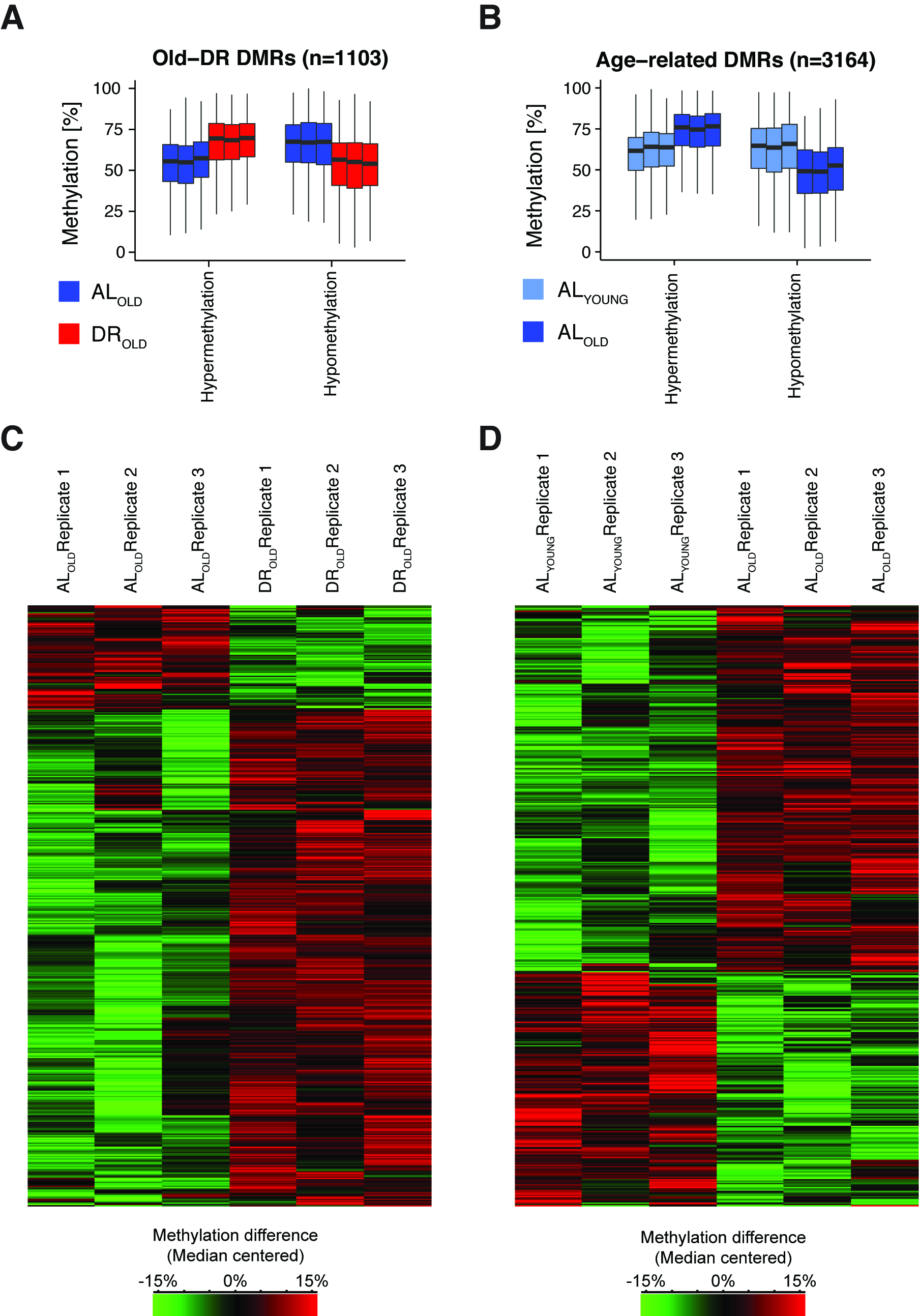
**

**Additional file 1: Fig. S11. Analysis of intra-replicate variance.** (A-B) Boxplot representation of the distribution of replicate-wise, quantified methylation values of Old-DR DMRs (A) and age-related DMRs (B) separated for hypo- and hypermethylation events. Bins previously defined based on merged replicate data were used to quantify bin-wise methylation values for each replicate. To account for the lower coverage per sample, methylation values were calculated if a bin contained 20 CpGs with at least one read in each replicate (n=1167422 bins after filtering). (C-D) Heatmaps representing per-bin normalised methylation values for replicate-wise, quantified methylation values of Old-DR DMRs (C) and age-related DMRs (D). All replicates display clear inter-replicate variance for the selected sets of DMRs, compared to their intra-replicate variance.
